# Oncogenic KRAS supports pancreatic cancer through regulation of nucleotide synthesis

**DOI:** 10.1038/s41467-018-07472-8

**Published:** 2018-11-23

**Authors:** Naiara Santana-Codina, Anjali A. Roeth, Yi Zhang, Annan Yang, Oksana Mashadova, John M. Asara, Xiaoxu Wang, Roderick T. Bronson, Costas A. Lyssiotis, Haoqiang Ying, Alec C. Kimmelman

**Affiliations:** 1 Division of Genomic Stability and DNA Repair, Department of Radiation Oncology, Dana-Farber Cancer Institute, Boston, MA 02215 USA; 2 Department of General, Visceral and Transplantation Surgery, RWTH Aachen University Hospital, Aachen, 52074 Germany; 3 Meyer Cancer Center, Weill Cornell Medicine, New York, NY 10065 USA; 4 Division of Signal Transduction, Department of Medicine, Beth Israel Deaconess Medical Center and Harvard Medical School, Boston, MA 02115 USA; 5 Rodent Histopathology Core, Harvard Medical School, Boston, 02115 MA USA; 6 Department of Molecular and Integrative Physiology, University of Michigan, Ann Arbor, MI 48109 USA; 7 Division of Gastroenterology, Department of Internal Medicine, University of Michigan, Ann Arbor, MI 48109 USA; 8 Department of Molecular and Cellular Oncology, The University of Texas MD Anderson Cancer Center, Houston, Texas 77030 USA; 9 Department of Radiation Oncology, Perlmutter Cancer Center, New York University School of Medicine, New York, NY 10016 USA

## Abstract

Oncogenic KRAS is the key driver of pancreatic ductal adenocarcinoma (PDAC). We previously described a role for KRAS in PDAC tumor maintenance through rewiring of cellular metabolism to support proliferation. Understanding the details of this metabolic reprogramming in human PDAC may provide novel therapeutic opportunities. Here we show that the dependence on oncogenic KRAS correlates with specific metabolic profiles that involve maintenance of nucleotide pools as key mediators of KRAS-dependence. KRAS promotes these effects by activating a MAPK-dependent signaling pathway leading to MYC upregulation and transcription of the non-oxidative pentose phosphate pathway (PPP) gene RPIA, which results in nucleotide biosynthesis. The use of MEK inhibitors recapitulates the KRAS-dependence pattern and the expected metabolic changes. Antagonizing the PPP or pyrimidine biosynthesis inhibits the growth of KRAS-resistant cells. Together, these data reveal differential metabolic rewiring between KRAS-resistant and sensitive cells, and demonstrate that targeting nucleotide metabolism can overcome resistance to KRAS/MEK inhibition.

## Abstract (web summary)

Pancreatic ductal adenocarcinoma (PDAC) cells display varying degrees of reliance on oncogenic KRAS. Here the authors show that KRAS-resistant PDAC cells maintain nucleotides synthesis through a KRAS-independent upregulation of the non-oxidative pentose phosphate pathway gene RPIA and that targeting nucleotide metabolism restore sensitivity to KRAS pathway inhibition.

## Introduction

Pancreatic ductal adenocarcinoma (PDAC) is estimated to become the second cause of cancer-related death by 2020^1^, with an expected 5-year survival rate of ~8%^2^. This poor prognosis is a consequence of late stage diagnosis, which limits surgical intervention, as well as resistance to conventional treatments such as chemotherapy and radiation^3^. The genetic events that drive pancreatic intraepithelial neoplasia (PanIN) formation and progression to PDAC are well known and have been validated in multiple mouse models. These involve mutations in tumor suppressor genes like *CDKN2A* (that encodes *INK4A* or *ARF*), *TP53* and *SMAD4*, as well as activation of the *KRAS* oncogene^4–6^. Despite the knowledge of the PDAC genetic signature, therapeutic efforts to inhibit the key oncogenic driver, KRAS, have been largely unsuccessful.

To facilitate survival and proliferation, tumor cells rewire their metabolism to maintain redox homeostasis and fuel anabolic pathways^7–9^. Indeed, work from our lab has shown a critical role for a novel glutamine-dependent pathway in the maintenance of redox balance and tumor growth in PDAC^10^. In addition, we and others have reported on the engagement of metabolic recycling and scavenging pathways in pancreatic cancer, which enable biosynthetic activity in a nutrient poor microenvironment^11–14^. Beyond extracellular scavenging, pancreatic cancer cells also engage metabolically with other cells in the tumor microenvironment^15^. For example, we have recently described a model underlining the importance of the microenvironment and its contribution to tumor metabolism in which pancreatic stellate cells secrete alanine that can be taken up by PDAC cells to fuel several catabolic and anabolic processes^9^.

Growing evidence supports that activation of several oncogenes such as KRAS and MYC can induce metabolic reprogramming^16^ and this opens the possibility of new therapeutic strategies, targeting the deregulated metabolism in cancer^17,18^. We previously generated an inducible KrasG12D (iKras) genetically engineered mouse model (GEMM), with which we revealed an essential role of Kras in tumor maintenance in vivo, and this functioned in part through the reprogramming of metabolism^19^. Kras promoted an increase in glucose uptake, shunting glucose intermediates into the hexosamine biosynthesis pathway (HBP) and the non-oxidative pentose phosphate pathway (PPP). This shift decoupled redox control from ribose synthesis, with redox regulated by malic enzyme 1 (ME1)^10^. The KRAS-driven metabolic changes were mediated by the RAF/MEK/ERK pathway, which results in upregulation of MYC and the transcriptional regulation of rate-limiting enzymes in glucose metabolism^19^.

This study drew attention to several potential therapeutic strategies, either by directly targeting MEK or the metabolic pathways over-activated in a mutant KRAS context. However, there were several aspects that remained to be defined. While it has become clear that tumors may have varying degrees of reliance on oncogenic KRAS^20,21^, it will be critical to understand how KRAS-dependence impacts metabolic rewiring and the associated vulnerabilities. Additionally, the context-specific dependence on KRAS-driven metabolic pathways remains to be determined. Here, we have characterized the differential metabolic profiles of KRAS-resistant and sensitive human PDAC cell lines and identified metabolic susceptibilities of KRAS-resistant cells. Additionally, we have shown that KRAS dependency is in large part due to its role in nucleotide biosynthesis. Overall, this work describes a mechanism by which KRAS mediates tumor survival and metabolic reprogramming in human PDAC and highlights potential therapeutic targets in the nucleotide biosynthesis pathway to overcome KRAS/MEK inhibitor resistance.

## Results

### Kras dependency correlates with differential metabolic rewiring

**Fig. 1:**
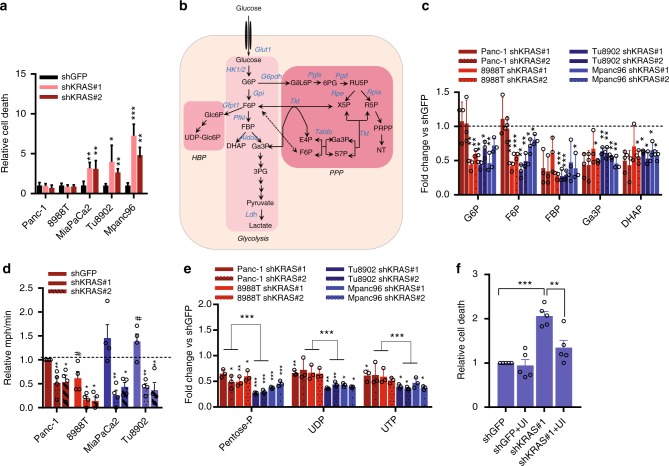
KRAS inhibition induces differential metabolic rewiring in PDAC cells. **a** KRAS inhibition impacts viability differentially after 48 h in complete media, as shown by trypan-blue exclusion assay. Error bars represent s.e.m. of 3 independent experiments. **b** Scheme representing the main enzymes and metabolites in the glycolytic and the pentose phosphate pathway (PPP). **c** Fold change of glycolytic intermediates after KRAS inhibition, each shRNA relative to its corresponding shGFP. Error bars represent s.d. of *n* = 3 technical replicates from independently prepared samples from individual wells. Significance determined for each shRNA vs. shGFP. G6P, glucose 6-phosphate; F6P, fructose 6-phosphate; FBP, fructose 1,6-bisphosphate; Ga3P, glyceraldehyde 3-phosphate; DHAP, dihydroxyacetone phosphate. **d** Extracellular acidification rate (ECAR) is decreased after KRAS depletion. All values normalized to cell number determined by crystal violet staining and fold change normalized to Panc-1 shGFP. Error bars represent s.e.m. of 4 independent experiments (* shows comparison to each cell line’s shGFP, # shows comparison to Panc1-shGFP). **e** Fold change of PPP intermediates and pyrimidines after KRAS inhibition, each shRNA normalized to its corresponding shGFP. Error bars represent s.d. of *n* = 3 technical replicates from independently prepared samples from individual wells. Significance determined for each shRNA vs. shGFP. Pentose-P, Pentose-phosphate; UDP, uridine diphosphate; UTP, uridine triphosphate. **f** Cell death analyzed by flow cytometry after KRAS depletion in Tu8902 cells in media supplemented with U (uridine) and I (inosine) at 1 mM, respectively. Bars represent relative fold change in cell death vs. shGFP (error bars show s.e.m of 5 independent experiments). For all panels, significance determined with *t-test*. *p < 0.05, **p < 0.01, ***p < 0.001

It has been previously reported that mutant KRAS cell lines could be classified as KRAS-dependent and independent based on their sensitivity to KRAS knockdown^20,21^. To investigate the role of KRAS in metabolic reprogramming, we initially interrogated a panel of human PDAC cell lines for differential sensitivity to KRAS depletion. We downregulated KRAS in PDAC cells using two lentiviral short hairpin RNAs (shRNAs) and we measured their ability to grow and form colonies in clonogenic assays. KRAS ablation impacted growth and clonogenic growth of all cell lines tested, but some cell lines (referred as KRAS-resistant) were still able to form colonies independent of KRAS expression (Supplementary Fig. 1a). Although KRAS inhibition decreased proliferation in all cell lines (Supplementary Fig. 1b), no induction of cell death was observed in KRAS-resistant lines (Fig. 1a). In contrast, KRAS depletion induced significant cell death in KRAS-sensitive cell lines (Fig. 1a).

We previously described that Kras activation in a PDAC GEMM orchestrates a metabolic program that consists in increased flux through glycolysis, non-oxidative PPP and HBP (Fig. 1b) through transcriptional control of key enzymes in these pathways^19^. To evaluate the metabolic profile in the set of human KRAS-resistant and sensitive cells, we performed targeted liquid chromatography-tandem mass spectrometry (LC-MS/MS) after KRAS depletion. Consistent with the role of KRAS in regulating glycolysis^18,19^, our metabolomic studies showed a decrease in several glycolytic intermediates including fructose bisphosphate (FBP), glyceraldehyde-3-phosphate (Ga3P) and dihydroxyacetone-phosphate (DHAP) in nearly all cell lines after KRAS extinction (Fig. 1c). To confirm the general decrease in glycolysis after KRAS inactivation, we measured the extracellular media acidification rate (ECAR) (Fig. 1d). KRAS depletion decreased ECAR in all cell lines consistent with the metabolomics data. These metabolic changes correlated with reduced expression of rate-limiting glycolytic genes (HK1, HK2) (Supplementary Fig. 1c). This is in agreement with the iKras GEMM^19^ suggesting that KRAS controls glucose metabolism in the majority of human PDAC cells by regulating the expression of glycolytic enzymes. Consistent with our initial findings, metabolites in the HBP (Supplementary Fig. 1d) were not differentially regulated in resistant vs. sensitive cells, suggesting that KRAS broadly supports both glycolysis and HBP.

The iKras GEMM highlighted the importance of incorporating glucose into the non-oxidative PPP for ribose and nucleotide biosynthesis^19^. Supporting this idea, KRAS extinction decreased pentose phosphate in all cell lines, but significantly more in sensitive cells (Fig. 1e). Consistent with the reduction in PPP intermediates, pyrimidine nucleotides like UDP and UTP dropped in all cell lines after shKras, but more significantly in sensitive cells (Fig. 1e). These results suggest a differential response to KRAS depletion in resistant vs. sensitive cells in the regulation of the PPP and nucleotide biosynthesis.

To confirm the importance of nucleotide metabolism, cell death was assessed in KRAS-depleted sensitive cells cultured in media supplemented with ribo and deoxyribonucleosides (Supplementary Fig. 1e). This combination of nucleosides was able to rescue cell death upon KRAS knockdown (Supplementary Fig. 1e). Furthermore, the addition of uridine, essential for synthesis of pyrimidines, and inosine, important for purine synthesis and salvage pathways, were also able to suppress cell death (Fig. 1f, Supplementary Fig. 1f–g). Interestingly, the ability of KRAS to support cell proliferation was not impacted by the addition of nucleosides indicating that other aspects of KRAS function are required for proliferation (Supplementary Fig. 1h). Together these data indicate that KRAS extinction induces a differential reduction in nucleotide synthesis in sensitive and resistant cells that leads to cell death in KRAS-addicted cell lines, and restoring nucleotide pools via supplementation can rescue survival. In a similar manner, iKras mouse PDAC cell lines derived from tumors that developed resistance to doxycycline-mediated loss of KrasG12D (escaper tumor lines, iKras−)^22^ displayed decreased glycolysis as shown by decreased glucose uptake/lactate excretion (Supplementary Fig. 2a). These lines are compared to iKras mouse PDAC cell lines derived from tumors that developed resistance to doxycycline-mediated KrasG12D control but were still dependent on Kras signaling (iKras+). Furthermore, the iKras- lines also showed a drop in glycolytic intermediates including DHAP, 3PG, G3P and PEP (Supplementary Fig. 2b) in steady state levels, and this was validated using 13C-labeled carbon tracing (Supplementary Fig. 2c). In contrast to the human knockdown studies, iKras− and iKras+cells had similar PPP metabolite levels, which may reflect an adaption to Kras independence (Supplementary Fig. 2d–e). To determine PPP activity, we calculated the flux of glucose carbon into RNA and found that Kras-dependent murine PDA lines (iKras+) exhibited, proportionally, a much greater utilization of the non-oxidative PPP (Supplementary Fig. 2f). These results further corroborate the correlation between Kras dependence and non-oxidative PPP utilization.

### The MAPK-MYC-RPIA pathway mediates KRAS resistance

**Fig. 2:**
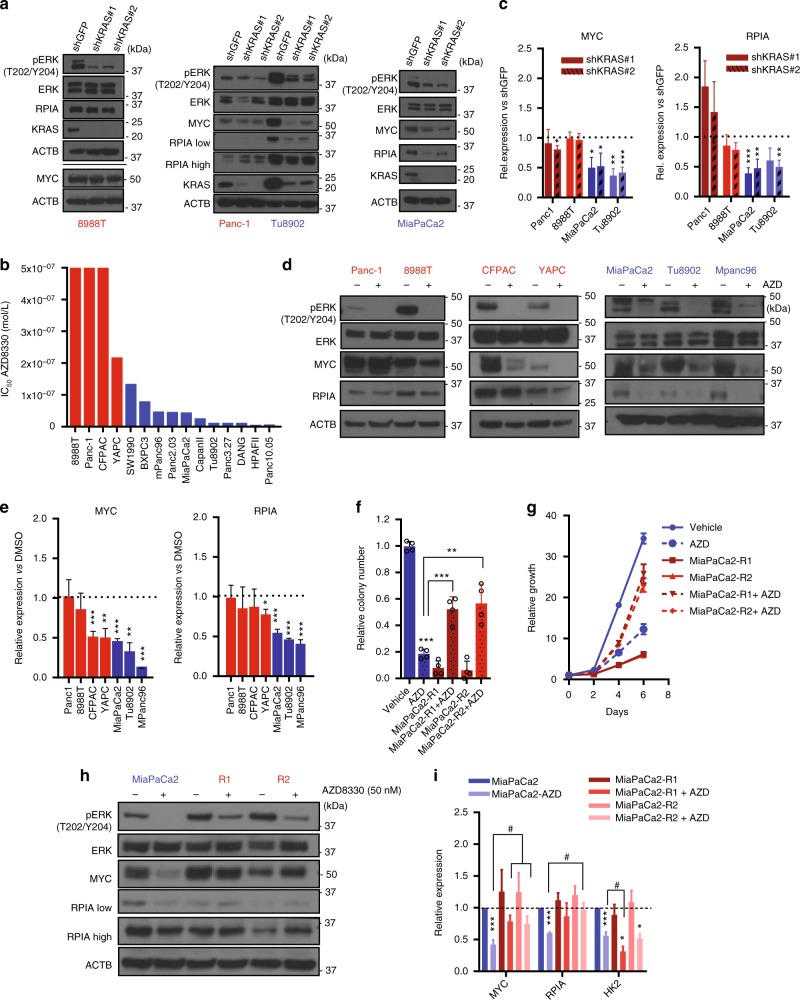
The MAPK-MYC-RPIA pathway defines resistance to KRAS and MEK inhibition. **a** Immunoblotting of human PDAC cell lines where KRAS was downregulated using 2 lentiviral short hairpin RNAs (shRNAs). **b** IC_50_ measurements (*y*-axis, IC_50_ mol/L) of PDAC cells (*x*-axis) treated with the MEK inhibitor AZD8330. **c** MYC and RPIA mRNA levels assessed by RT-qPCR in cells were KRAS was downregulated. Values are normalized to β-actin and each shRNA normalized to their corresponding shGFP. Error bars represent s.e.m. of at least three independent experiments. **d** Relative expression of proteins assessed by western blot in human PDAC cells treated with AZD8330 for 16 h (left: KRAS-resistant, right: KRAS-sensitive). **e** Relative mRNA levels of MYC and RPIA in the presence of AZD8330 for 16 h. All values normalized to β-actin and relative to DMSO treated cells. Error bars represent s.e.m. of independent experiments (CFPAC *n* = 5, YAPC, 8988 T *n* = 4, Panc-1, MiaPaCa2, Tu8902, MPanc96 *n* = 3). **f** MiaPaCa2 cell lines resistant to MEK inhibition were generated (AZD8330 = 50 nM). Colony number was normalized to MiaPaCa2 with vehicle. Error bars indicate s.d. of 2 technical replicates in 2 independent experiments. **g** Relative growth normalized to day 0. Error bars represent s.d. of technical replicates (one representative of 3 experiments). **h** Immunoblot of MiaPaCa2-resistant cells cultured with AZD8330 for 16 h maintained MYC and RPIA expression compared to treated MiaPaCa2 cells. **i** mRNA expression levels of MYC, RPIA and HK2 in MiaPaCa2 and MiaPaCa2 resistant cells after 16 h of AZD8330 exposure. Values are normalized to β-actin and each condition normalized to MiaPaCa2 with vehicle. Error bars represent s.e.m of at least three independent experiments (*shows comparison to each cell’s line control, # shows comparison to MiaPaCa2-AZD). For all panels, significance determined with *t-test*. *p < 0.05, **p < 0.01, ***p < 0.001

KRAS exerts its effects by activation of several canonical downstream pathways. Several studies have identified the MAPK pathway as the main mediator of KRAS tumorigenic effects in PDAC^19,23^. In agreement with this, we confirmed that KRAS depletion decreased Erk phosphorylation (Thr 202/Tyr 204) (Fig. 2a) with a minor impact on pAkt (Ser473)/Akt levels (Supplementary Fig. 3a). Indeed, MEK inhibition correlated with the KRAS dependence pattern, with KRAS-resistant cells showing the highest IC_50_ both for AZD8330 (Fig. 2b), as well as a second MEK inhibitor, trametinib (Supplementary Fig. 3b–c). Further analysis of downstream mediators after KRAS depletion, revealed a downregulation of Myc levels mainly in sensitive cells, at the RNA and protein levels (Fig. 2a–c). We had shown previously that RPIA is a MYC-regulated gene downstream of oncogenic KRAS, in the non-oxidative PPP^19,24^, essential for ribose biosynthesis^25^ and crucial mediator of PDAC tumorigenesis in mice^19^. Given the differences in nucleotide metabolism in resistant and sensitive cells, we investigated RPIA levels after KRAS extinction. KRAS inhibition reduced RPIA expression in all sensitive cell lines, but had minimal impact in the resistant lines (Fig. 2a–c). These results suggest that resistance to KRAS inhibition may be mediated by convergent mechanisms that ultimately lead to maintenance of RPIA expression in order to maintain nucleotide pools. Similar results were obtained with MEK inhibition, showing minimal changes in RPIA expression in the resistant lines both with AZD8330 (Fig. 2d, e) and trametinib (Supplementary Fig. 3e, f). Interestingly, we discovered a subset of resistant cell lines that are able to maintain RPIA expression despite decreasing MYC levels, further reinforcing the role of RPIA in mediating KRAS resistance (Fig. 2d, e).

While MEK inhibition exhibited similar effects as Kras knockdown in many regards, a clear distinction was that MEK inhibition induced cytostasis instead of cell death (Supplementary Fig. 3d). As previously reported^26^, MEK inhibition induced compensatory activation of the PI3K pathway as evidenced by AKT activation (Supplementary Fig. 3g) in sensitive lines. In contrast, KRAS knockdown in sensitive lines did not result in compensatory AKT activation (Supplementary Fig. 3a). We therefore speculated that activation of the PI3K pathway in the setting of MEK inhibitor treatment could explain the differential cytostatic effect observed with MEK inhibition compared to the shKRAS. Consistent with this, concurrent inhibition of AKT and MEK increased cell death in sensitive cells (Supplementary Fig. 3h).

To further understand the role of the MEK-MYC-RPIA axis in KRAS/MEK dependency, we generated two resistant clones of the MiaPaCa2 cell line that were able to grow and form colonies in the presence of AZD8330 (Fig. 2f, g). In contrast to the parental line, the newly generated resistant cells sustained MYC and RPIA levels after MEK inhibition (Fig. 2h, i), while HK2 expression was not recovered (Fig. 2i). Together these data suggest that expression of MYC and RPIA mediate resistance to MEK inhibitors and that resistance is not likely due to a global reactivation of KRAS-dependent metabolism.

**Fig. 3:**
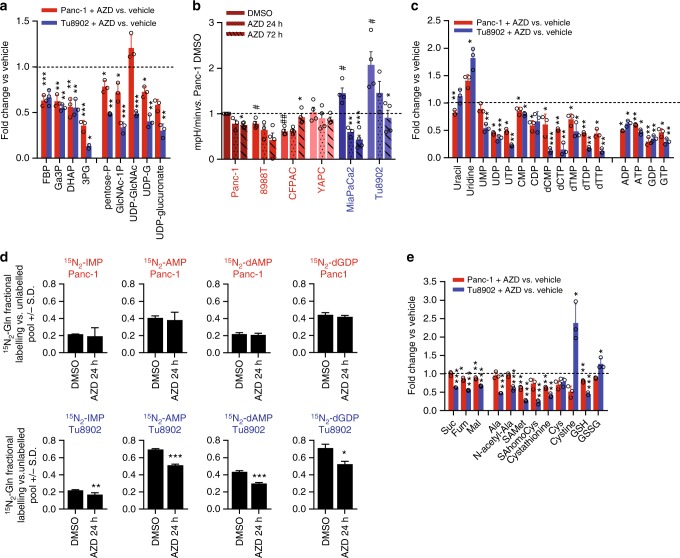
MEK inhibitor-induced metabolic reprogramming is comparable to shKras. **a** For all metabolomics experiments in this figure, cells were treated with AZD8330 (50 nM) for 72 h in media containing glucose 25 mM and glutamine 4 mM. Values are represented as fold change vs. DMSO-treated cells for glycolysis, PPP and HBP metabolites. Error bars represent s.d. of *n* = 3 technical replicates from independently prepared samples from individual wells. Significance determined for each cell line treated with AZD8330 vs. vehicle**. b** Quantification of Extracellular Acidification Rate (ECAR) normalized to cell number determined by crystal violet staining. All values relative to Panc-1 DMSO, error bars ± s.e.m. of independent experiments (* shows comparison to each cell line’s control (DMSO), # shows comparison to Panc1-DMSO). **c** Fold change of metabolites in the pyrimidine and purine pathways after AZD8330 treatment. UMP, uridine monophosphate; CDP, cytidine diphosphate, CTP, cytidine triphosphate; dCTP, deoxy CTP; dTTP, deoxy thymidine triphosphate; IMP, inosine monophosphate; AMP, adenosine monophosphate; dATP, deoxy adenosine triphosphate; GDP, guanosine diphosphate. Significance determined for each cell line treated with AZD8330 vs. vehicle. **d** Tracing experiments in Panc-1 and Tu8902 cells treated with AZD8330 (50 nM) in medium containing stable isotope-labeled glutamine (Amide-15N) for 24 h to label incorporation into the purine ring. Bars show fractional labeling vs. unlabeled pool, error bars indicate ± s.d. of *n* = 3 technical replicates from independently prepared samples from individual wells. **e** Fold change of metabolites in the tricarboxylic acid (TCA) cycle, transaminase and redox pathways after AZD8330 treatment, error bars indicate ± s.d. of *n* = 3 technical replicates from independently prepared samples from individual wells. Significance determined for each cell line treated with AZD8330 vs. vehicle. Ala, alanine; SAHomoCys, S-adenosyl-L-homocysteine; GSH, reduced glutathione; GSSG, oxidized glutathione. For all panels, significance determined with *t-test*. *p < 0.05, **p < 0.01, ***p < 0.001

Also in agreement with the shKras-induced metabolic changes, MEK inhibition for 72 h decreased glycolysis, seen as a drop in glycolytic metabolites in resistant and sensitive cells (Fig. 3a), as well as ECAR (Fig. 3b) more prominently in sensitive cells. MEK inhibition decreased nucleotide synthesis (purine and pyrimidines) in sensitive and resistant lines at 72 h (Fig. 3c) but more profoundly in sensitive cells. These differences were enhanced when cells were treated with a MEK inhibitor for 24 h in lower glucose/glutamine conditions, showing a significant decrease in glycolysis (Supplementary Fig. 4a), PPP (Supplementary Fig. 4b) and purines/pyrimidines (Supplementary Fig. 4c) only in sensitive cells. The decrease in nucleotide biosynthesis was confirmed by N15-Glutamine tracing studies, which showed a trend towards lower incorporation in sensitive cell lines as compared to resistant for pyrimidines (Supplementary Fig. 4d) and more significantly purines (Fig. 3d) upon MEK inhibition. This pattern correlated with selective drop in glycolytic and PPP gene expression after AZD8330 (Supplementary Fig. 4e) and trametinib (Supplementary Fig. 3f) treatment in sensitive lines. Finally, mitochondrial metabolism was also differentially impacted in sensitive cells, as shown by decrease in TCA cycle/redox intermediates (Fig. 3e, Supplementary Fig. 4f), respiration (Supplementary Fig. 4g), and GSH/GSSG ratio (Supplementary Fig. 4h).

**Fig. 4:**
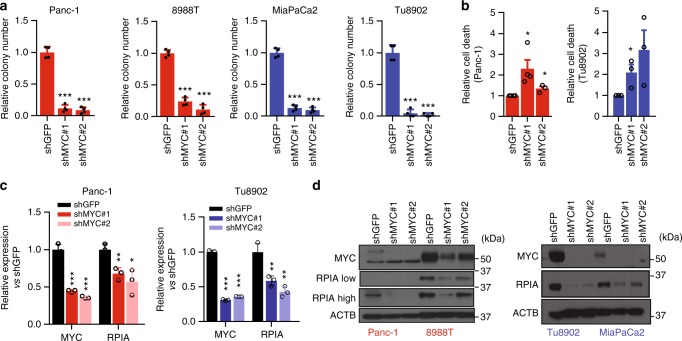
MYC is required for survival of PDAC cells. **a** Clonogenic growth of PDAC cells expressing a control shRNA (shGFP) or two independent shRNAs targeting MYC. Error bars indicate s.d. of 2 technical replicates in 2 independent experiments. **b** Evaluation of cell death induction after MYC down-regulation using Annexin V-FITC and propidium iodide flow cytometry. Relative fold change in cell death of averaged experiments (error bars show s.e.m). **c** Relative mRNA levels of MYC and RPIA after MYC inhibition. Values are normalized to β-actin. Error bars represent s.d. of three technical replicates (1 representative of 3 experiments). **d** Immunoblot of PDAC cells expressing shGFP or shMYC shRNAs. For all panels, significance determined with *t-test*. *p < 0.05, **p < 0.01, ***p < 0.001

Finally, to confirm the role of MYC as a mediator of KRAS resistance, we suppressed MYC expression using RNAi. MYC depletion decreased the survival and proliferation of all cell lines (Fig. 4a, b, Supplementary Fig. 5a–b) but it had a cytostatic effect in non-transformed cell lines (Supplementary Fig. 5a, c), confirming its essential role in PDAC survival. MYC ablation reduced RPIA levels in resistant and sensitive cells, both at the RNA (Fig. 4c) and protein levels (Fig. 4d). Together these data suggest that expression of MYC and RPIA mediates resistance to MEK inhibitors.

### RPIA inhibition sensitizes KRAS-resistant cells

**Fig. 5:**
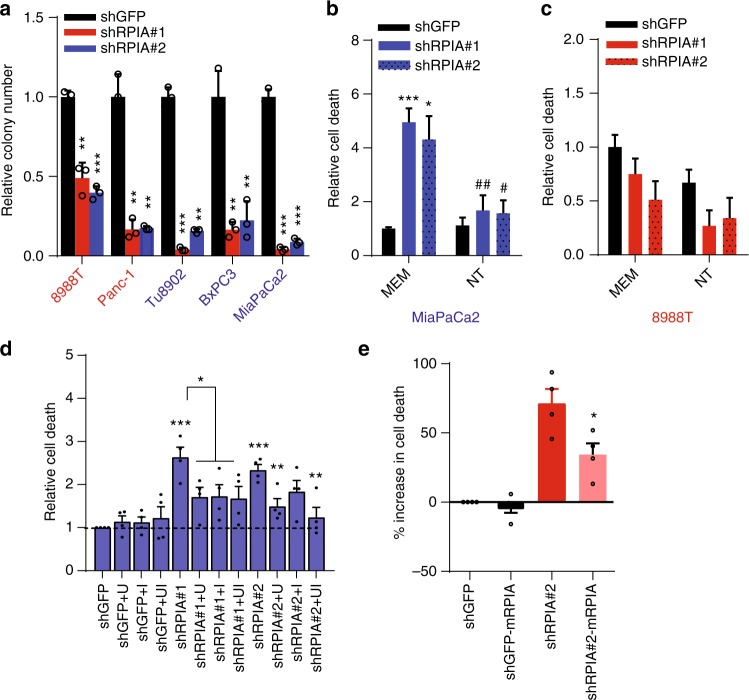
RPIA and nucleotide biosynthesis are essential for PDAC cell survival. **a** RPIA was targeted using two different shRNAs. Clonogenic growth was assessed in several human PDAC cells compared to the control shGFP. Error bars ± s.d., triplicate wells of a representative experiment (8988 T, MiaPaCa2 *n* = 4; Panc-1, BxPC3 *n* = 3; Tu8902 *n* = 2). **b** and **c** Cells depleted of RPIA were cultured in MEM or MEM containing nucleosides (10 mg/L or ≈0.04 mM, each) and cell death was assessed by trypan blue exclusion. Error bars represent s.e.m. of 3 independent experiments (* shows comparison to shGFP, # shows comparison to shRPIA in MEM). **d** Cell death analyzed by flow cytometry after RPIA depletion in MiaPaCa2 cells in media supplemented with U (uridine) and I (inosine) alone or in combination (1 mM, respectively). Bars represent relative fold change in cell death vs. shGFP of 4 averaged independent experiments (error bars show s.e.m). **e** Cell death analyzed by flow cytometry after RPIA depletion and mRPIA expression in MiaPaCa2 cells. Bars represent increase in cell death as compared to shGFP (error bars show s.e.m of 4 independent experiments). For all panels, significance determined with *t-test*. *p < 0.05, **p < 0.01, ***p < 0.001

Given the limited pharmacological strategies available to inhibit KRAS and the resistance we observed to MEK inhibitors, targeting of downstream metabolic pathways may be an attractive strategy to inhibit PDAC growth, particularly in tumors that have de novo or acquired resistance to MEK or KRAS inhibition. Our results highlighted a pathway downstream of KRAS, leading to RPIA expression that was critical for mediating resistance to MEK inhibition. RPIA depletion reduced growth and clonogenic survival of both resistant and sensitive cell lines (Fig. 5a, Supplementary Fig. 5d) proving that the PPP is an essential pathway to maintain PDAC growth. RPIA deletion induced cell death in most of the cell lines tested (Fig. 5b, Supplementary Figure 5e) although to a lesser extent than Kras depletion. On the other hand, RPIA deletion didn’t induce cell death (Supplementary Fig. 5c) or impair growth (Supplementary Fig. 5d) in non-transformed cell lines (IMR-90 and hPSC), indicating there might be an optimal therapeutic window for treating PDAC with RPIA inhibition. Interestingly, one of the KRAS-resistant lines (8988 T) was more resistant to RPIA inhibition (Fig. 5a, Supplementary Fig. 5d) than other lines, suggesting that other compensatory mechanisms might be acting in this particular cell line. CRISPR deletion of RPIA (Supplementary Fig. 5f) produced similar data to the shRNA studies (Supplementary Fig. 5g).

Consistent with the importance of nucleotide metabolism in this phenotype, cell death induced by RPIA downregulation was attenuated when shRPIA cells were cultured in a media supplemented with nucleosides (Fig. 5b, c). Additionally, supplementation of Uridine and Inosine alone or in combination, was also able to rescue cell death (Fig. 5d). Nucleotides were also able to partially recover colony formation in a KRAS-sensitive and resistant cell line after RPIA CRISPR KO (Supplementary Fig. 5h) or shRPIA (Supplementary Fig. 5i). The rescue of the RPIA depletion with nucleotides was more pronounced than that seen in rescuing shKras cells, further highlighting the broader functions of oncogenic KRAS. Finally, overexpression of a mouse RPIA cDNA was able to rescue cell death in shRPIA cells (Fig. 5e, Supplementary Figure 5j), confirming an on-target inhibition of the protein. Together, these data reinforce the importance of the non-oxidative PPP in maintaining nucleotide pools and survival in PDAC cells. Also, it highlights RPIA as an attractive therapeutic target in PDAC tumors that are resistant to MEK inhibitors.

### Inhibiting pyrimidine synthesis as a strategy to target PDAC

Our metabolomic studies showed a differential decrease in nucleotides after KRAS depletion (Fig. 1e) and MEK inhibition (Supplementary Fig. 4c) between sensitive and resistant cells, suggesting that inhibition of other nucleotide biosynthetic pathways could sensitize resistant cells in a similar fashion as PPP blockade.

While RPIA may be a potential therapeutic target, there are currently no available inhibitors. DHODH is an enzyme in the pyrimidine biosynthesis pathway that enables the conversion of dihydroorotate to orotate. Although nucleotide biosynthetic processes take place in the cytoplasm, DHODH is located in the inner mitochondrial membrane, where it associates with complex II and III of the electron transport chain and participates in ubiquinone reduction^27^. Therefore, DHODH inhibition could dually target respiration and nucleotide synthesis, two of the important pathways that appear necessary in PDAC^9,10,14,28,29^.

**Fig. 6:**
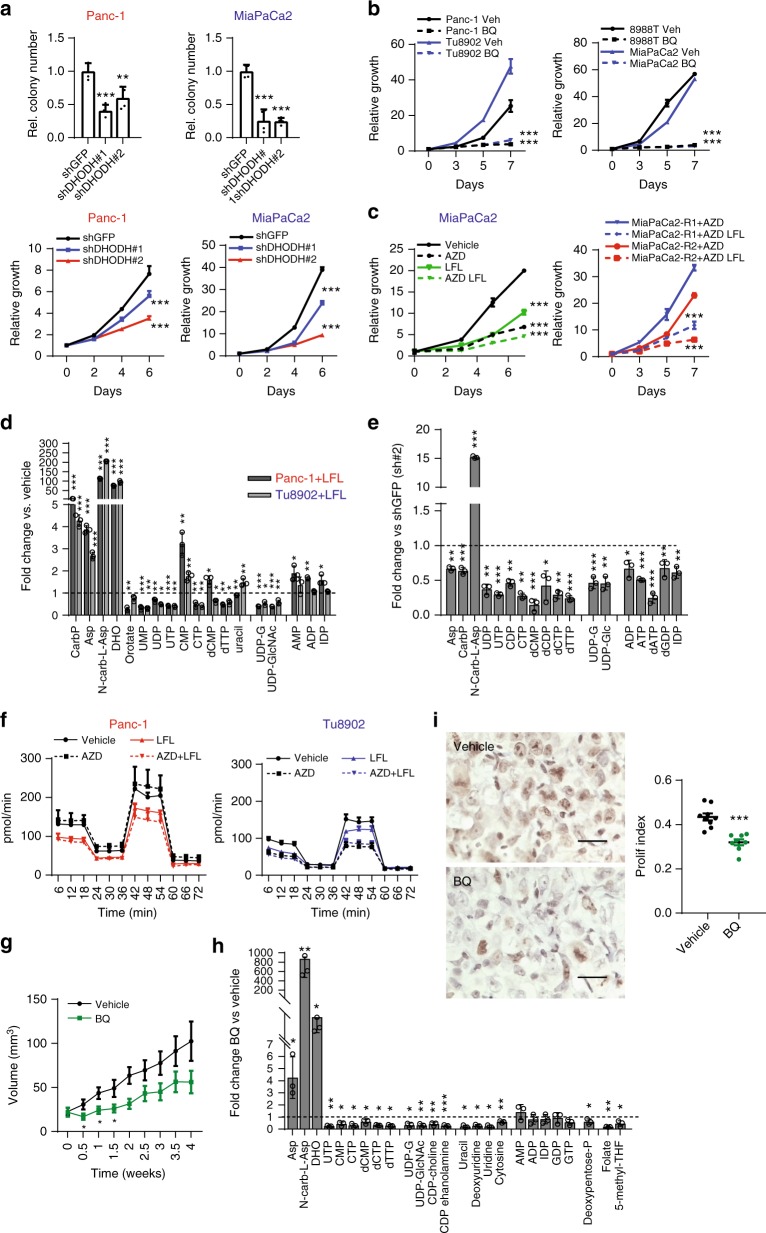
DHODH inhibition blocks pyrimidine synthesis and sensitizes KRAS-resistant cells in vitro and in vivo. **a** Clonogenic growth (top) and relative proliferation (bottom) of Panc-1 and Tu8902 cells assessed after DHODH depletion using two different shRNAs. Relative colony number is normalized to shGFP control (error bars indicate s.d. of 2 technical replicates in 2 independent experiments). **b** Cell growth curves for Brequinar treated cells (50 μM). Values are normalized to Day 0. Error bars represent s.d. of triplicate wells from a representative experiment (*n* = 2). **c** MiaPaCa2 (left) and MiaPaCa2-resistant cells (right) decreased growth when leflunomide (100 μM) was added to media containing AZD8330. Error bars represent s.d. of quadruplicate wells from a representative experiment (*n* = 3). **d** LC-MS/MS metabolomic analysis of Panc-1 and Tu8902 cells treated with leflunomide (100 μM) for 16 h in 25 mM glucose/4 mM glutamine. Fold change relative to vehicle (methanol). Error bars represent s.d. of *n* = 3 technical replicates from independently prepared samples from individual wells. Significance determined for each cell line treated with AZD8330 vs. vehicle. Carb, carbamoyl; CMP/CTP, cytidine mono/triphosphate; UDP-G, UDP-glucose; UDP-GlcNAc; UDP-N-Acetyl-Glucosamine; IDP, Inosine diphosphate. **e** Fold change of metabolites in Tu8902 cells after DHODH inhibition (sh#2) relative to shGFP. Error bars represent s.d. of *n* = 3 technical replicates from independently prepared samples from individual wells**. f** Leflunomide (50 μM) decreases OCR in Panc-1 (left) and Tu8902 cells (right). AZD, AZD8330; LFL, leflunomide. Error bars show s.d. of 4 independent wells from a representative of 3 experiments. **g** Tumor growth is impaired after brequinar treatment (50 mg/kg). Error bars represent s.e.m. for 10 tumors per condition,*t*-tests were performed at each time point. **h** LC-MS/MS metabolomic analysis of tumors extracted after 4 weeks of treatment. Fold change of BQ-treated relative to vehicle. Error bars represent s.d. of *n* = 3 tumors per group. **i** BQ treatment decreases KI67 expression in treated mice vs. vehicle (left): representative field (40×) of 5 quantified fields per animal (8 mice per group, scale bar = 25 µm), (right): proliferation index calculated as number of positive cells vs. total cells, error bars show s.e.m. For all panels, significance determined with *t-test*. *p < 0.05, **p < 0.01, ***p < 0.001

In order to investigate the potential role of DHODH inhibition in PDAC growth, DHODH was depleted in our panel of PDAC cell lines using shRNAs. DHODH inhibition (Supplementary Fig. 6a) decreased growth and clonogenic survival of both resistant and sensitive cells (Fig. 6a, Supplementary Fig. 6b) with only minor effects in non-transformed cells (Supplementary Fig. 6c, d). These effects were reproduced with brequinar (Fig. 6b) and leflunomide (Supplementary Fig. 6e), two pharmacological inhibitors of DHODH^30,31^. Furthermore, leflunomide also was effective in inhibiting growth of the two MiaPaCa2 MEKi-resistant cell lines (Fig. 6c). Additionally knockdown of DHODH, as well as RPIA inhibited 3D growth of PDAC spheroids (Supplementary Figure 6f).

To assess the metabolic effects of DHODH inhibition, we performed metabolomic studies in cells (Fig. 6d) treated with leflunomide. Leflunomide efficiently blocked DHODH activity, leading to an accumulation of upstream intermediates like dihydroorotate, N-carbamoyl aspartate, carbamoyl phosphate and aspartate, and a drop in downstream metabolites like orotate, uridine-derived nucleotides (UMP, UDP, UTP) and other pyrimidines (CTP, dTTP). DHODH inhibition did not decrease purines (Fig. 6d) but had significant effects on pyrimidine biosynthesis (Fig. 6d). There were also decreases in some upper glycolytic intermediates with DHODH inhibition (Supplementary Fig. 6g). Metabolomic analysis of shDHODH cells also confirmed the block in pyrimidine synthesis (Fig. 6e) but this was accompanied by decreases in purines (Fig. 6e) and glycolytic intermediates (Supplementary Fig. 6h), suggesting potential adaptive responses to a broader long-term effect of DHODH depletion seen with shRNAs as compared to acute pharmacological inhibition. Additionally, genetic ablation of DHODH, a key structural component in the ETC, would have potential scaffold effects not seen with pharmacologic inhibitors.

DHODH inhibition has been shown to decrease mitochondrial membrane potential and increase ROS^27^. To test the consequences of DHODH inhibition on mitochondrial metabolism, we measured OCR after leflunomide treatment. Leflunomide efficiently blocked basal and maximal respiration in both resistant and sensitive cell lines after 16 h of treatment (Fig. 6f). These differences were less evident when cells were incubated at shorter timepoints (1, 3, 6 h), suggesting that leflunomide’s effect on OCR was a consequence of several aspects including impaired pyrimidine synthesis and direct mitochondrial function (Supplementary Fig. 6i). Consistent with the lower levels of respiration after DHODH inhibition, several metabolites in the TCA cycle were also decreased after leflunomide treatment (Supplementary Fig. 6g) and shDHODH expression (Supplementary Fig. 6h). Also, in agreement with DHODH’s role in maintaining redox balance^27^, the GSH/GSSG ratio dropped after leflunomide treatment (Supplementary Fig. 6j) and DHODH depletion (Supplementary Fig. 6k). Finally, we assessed the potential anti-tumor effects of DHODH inhibition in a PDAC tumor model. Leflunomide has been previously tested in vivo, resulting in weight loss and lethargy and was poorly tolerated^32^. Therefore, we used brequinar (BQ), a more potent and specific inhibitor of DHODH which has shown efficacy in an AML model^32^. BQ was dosed once every other day, a pattern that showed minimal toxicity in mice (Supplementary Fig. 7a), with no signs of anemia (Supplementary Fig. 7b) or splenomegaly (Supplementary Fig. 7c). To test the efficacy of DHODH inhibition by BQ in vivo, we performed metabolomics of pancreata of treated mice (50 mg/kg) vs. vehicle controls 4 h after dosing, which is the time the drug reaches maximal concentration^33^. DHODH was efficiently blocked as shown by an accumulation of N-carbamoyl-aspartate and a drop in CTP and CDP-choline (Supplementary Fig. 7d, left) with minor changes in glycolysis (Supplementary Fig. 7d, right).

Given the on-target pharmacodynamic (PD) results, we next assessed the efficacy of BQ in PDAC xenografts using a KRAS-resistant line. BQ-treated mice showed a significant reduction in tumor volume at early and intermediate time-points, with two mice showing complete regressions of tumors (Fig. 6g, Supplementary Fig. 7e). However, the anti-tumor effect was diminished by the end of the experiment (Fig. 6g, Supplementary Fig. 7e), suggesting there are adaptive mechanisms.

In order to investigate the compensatory mechanisms being activated after long-term treatment, we performed metabolomic analysis of tumors at experimental end point (long-term) and compared it to mice dosed twice with BQ (short-term). The short-term treatment induced accumulation of pyrimidine precursors like aspartate, N-carbamoyl-aspartate and dihydroorotate (Supplementary Fig. 7f), which was more pronounced in the long-term treatment (Fig. 6h). Pyrimidines were downregulated in the treated group while purines were not affected (Fig. 6h), showing an on-target inhibition of DHODH throughout the course of the experiment. Other metabolites in glycolysis, TCA cycle and fatty acid metabolism (Supplementary Fig. 7g) were generally downregulated in BQ-treated mice, suggesting a slowed proliferation in this group. We assessed all tumors for expression of cleaved-caspase 3 and Ki67 to measure apoptosis and proliferation. Although we found no induction of apoptosis (Supplementary Fig. 7h), tumors from BQ-treated mice showed decreased proliferation (Fig. 6i). Finally, we observed a decrease in collagen (Supplementary Fig. 7i) and α-SMA (Supplementary Fig. 7j) in BQ-treated mice, suggesting BQ treatment might also impact the tumor microenvironment. Additionally, our data showed no recovery in the pyrimidine pools after BQ treatment along with a slower proliferation rate, which suggests the residual nucleotide pools might be enough to sustain a residual reduced proliferation rate.

## Discussion

Here, we describe a metabolic reprogramming that maintains nucleotide biosynthesis to support resistance to KRAS or MEK inhibition. In fact, targeting the PPP via RPIA depletion or inhibiting pyrimidine biosynthesis directly through DHODH shows activity in all human PDAC cell lines tested. We propose that targeting ribose or pyrimidine synthesis is an attractive therapeutic strategy even in tumors that have developed resistance to KRAS/MEK inhibition.

These studies also inform us regarding the metabolic heterogeneity of human PDAC tumors. In agreement with the iKras mouse model which is a highly Kras-dependent tumor^23^, we have confirmed that KRAS acts mainly through the MAPK pathway to support metabolism and growth. However, unlike the iKras GEMM, KRAS/MEKi-resistant cells are able to sustain MYC and/or RPIA expression downstream of MEK in order to maintain nucleotide synthesis. This becomes clear in the subset of intermediately KRAS-resistant cell lines originally classified as slow proliferating^34^, which downregulate MYC, but still maintain RPIA expression. Suppression of MYC or RPIA mediates cell death in most resistant and sensitive cell lines.

Our metabolomic studies suggest that either suppressing KRAS expression or inhibiting MEK/MAPK pharmacologically depletes nucleotides to a greater degree in sensitive lines than in resistant ones. Not unexpectedly, there are some differences between genetic inhibition of KRAS as compared to pharmacologic inhibition of MEK. These are likely a consequence of the fact that KRAS has multiple other effects in addition to MEK/MAPK activation, as well as to compensatory activation of additional signaling pathways in response to MEK inhibition, such as PI3K. The common metabolic feature both in human and mouse KRAS/MEK resistant cells is their ability to maintain nucleotide synthesis, which is important to maintain survival. This mechanism has been recently proven to be crucial in mediating resistance to other drugs like gemcitabine^35^. RPIA is an enzyme that catalyzes the conversion between ribose-5-phosphate and ribulose-5-phosphate and it has been implicated in hepatocarcinogenesis^36^. RPIA deletion in our model impacts purine/pyrimidine synthesis, leading to cell death and overcoming the resistance mechanism described previously.

Unfortunately, there are only a few available PPP inhibitors targeting genes in the oxidative arm (6-aminonicotinamide) or the non-oxidative arm (oxythiamine, TKT) and these lack potency and specificity. Given the dynamic nature of the non-ox PPP and its intertwining with glycolysis, it may be more prone to compensatory effects. Therefore, inhibition of enzymes further downstream in the pathway could result in more potent anti-tumor effects. DHODH is a particularly attractive target in the pyrimidine pathway and its mitochondrial location has proven to be important in mediating conversion of DHO to orotate together with reduction of ubiquinone to ubiquinol^32^. Therefore, DHODH couples nucleotide synthesis to respiration, targeting two of the pathways that are critical in PDAC. DHODH’s expression or activity is increased in several cancers^37^ and in the last few years there has been growing interest about its potential as therapeutic target in cancers such as AML^32^, BRAF mutant melanoma^38^, triple negative breast cancers^39^ and PTEN mutant tumors^40^. Leflunomide is an FDA-approved drug used in the clinic to treat rheumathoid arthritis^41^ and is a weak and non-specific DHODH inhibitor^42^. However, it has proven to be toxic in vivo at doses needed to inhibit DHODH^32^. On the other hand, Brequinar is a more potent and specific inhibitor of DHODH with proven efficacy when dosed in an intermittent pattern that allows periods of nucleotide depletion in a model of leukemia^32^. Brequinar’s effects in vitro were more potent than leflunomide and it showed activity as a monotherapy in a xenograft model of human PDAC. Despite initial activity and even some complete responses, tumors ultimately progressed at a slower but sustained rate with BQ treatment. We showed that BQ-treated tumors didn´t upregulate other metabolic pathways and showed lower activation of proliferation markers. It remains to be evaluated the relevance of the effects observed on the tumor microenvironment, as well as developing effective combinatorial therapeutic strategies. Furthermore, DHODH and RPIA inhibition was well tolerated in non transformed cells, suggesting that these pathways may have potential for therapeutic inhibition although more in vivo studies should be performed to conclusively determine this.

Overall, our work describes a differential metabolic rewiring in KRAS-resistant and sensitive cells, which is regulated by the MAPK-MYC-RPIA pathway. We identified nucleotide metabolism as a key mediator of KRAS resistance and we propose targeting enzymes in the PPP or the pyrimidine biosynthesis pathway to overcome resistance to MEK inhibitors and ultimately to future KRAS inhibitors that are in various stages of development.

## Methods

### Cell culture

Cell lines were obtained from ATCC and DSMZ. Establishment and growth conditions of immortalized HPDE cells were described elsewhere^43,44^ as well as hPSC^45^. Cells were grown in DMEM (Life Technologies 11965) or IMDM (CFPAC, ThermoFisher 12440053) with 10% FBS and 1% Pen/Strep (Life Technologies 15140). Mycoplasma was routinely tested by PCR. PDAC cell lines were maintained in a centralized bank and authenticated by assessment of cell morphology as well as STR fingerprinting. Mpanc96 was used as an additional KRAS/MEKi sensitive cell line based on prior literature. Subsequently, it was determined that this line is the same as ASPC1. We have verified this via STR fingerprinting. As we originally received the line as Mpanc96, we elected to use this nomenclature when describing the line.

MiaPaCa2 clones resistant to AZD8330 were generated by two different methods: MiaPaca2-R1 was cultured in slowly increasing concentration of the drug (5 to 50 nM) while MiaPaCa2-R2 was cultured immediately in 50 nM. 50 nM is sufficient to inhibit Erk phosphorylation in all cell lines with no changes in total Erk protein levels (Fig. 2d).

Nucleotide rescue experiments were approached with two different methods. Cells were grown in MEM (Sigma, D5030) or MEM containing a pool of nucleosides (MEM alpha, Life Technologies 12571). In both cases, glucose and glutamine concentration was adjusted to 25 mM and 4 mM, respectively, to keep consistent with DMEM. Alternatively, DMEM was supplemented with individual nucleosides (1 mM): uridine (Sigma U3003), inosine (Sigma, I4125) or the combination of both, during the selection process.

### Cell proliferation assays

Cells were plated in 24-well plates at 3000–5000 cells per well. The day after plating, cells were treated with AZD8330, trametinib, leflunomide or brequinar. Media was not refreshed for the duration of the assay. Cells were fixed in 10% formalin at the indicated time points and stained with 0.1% crystal violet. Dye was extracted with 10% acetic acid, and relative proliferation was determined by measuring OD at 595 nm. For 3D suspension experiments, 1000 cells were seeded in ultra-low attachment surface 96-well plates (Corning Costar #7007). Cell growth was monitored 5 days later by adding 50 µl of CellTiter-Glo reagent (Promega), mixing, covering and placing in a plate shaker for 10 min to ensure cell lysis prior to assessment of luminescent signal. Data was normalized to day 0.

### Clonogenic survival assay

Cells were plated in 6 cm plates at 500 cells per dish. The day after plating, drugs were added at the indicated concentrations. In case of nucleotide rescue, these were maintained in the media for the course of the experiment since selection. After 7 days, cells were fixed with 80% methanol and stained with 0.2% crystal violet. Colonies were counted and the surviving fraction was calculated using the plating efficiency.

### IC_50_ assay

Cells were plated in 96 well-plates at 2000 cells per well. The day after plating cells were treated by serial dilution of AZD8330 or trametinib. After 72 h, viability was measured using Cell-Titer Glo (Promega G7572) assay. Luminescence was measured on a POLARstar Omega plate reader. IC_50_ was calculated using GraphPad Prism non-linear regression.

### Cell death assays

*Trypan-blue exclusion*. shKras and shRPIA cells were plated in 6-well plates at 50% confluency with or without nucleosides. 48 h after plating, cells were trypsinized and resuspended in their own media. Trypan blue (Thermo-scientific 15250061) positive cells were counted with a haematocytometer and percentage of cell death was normalized to shGFP cells. *Flow cytometry analysis of apoptosis*. Cells were plated and trypsinized after 48 h, as described. Cells were stained with Annexin-FITC and propidium iodide (PI) for 15 min (BD Biosciences 556547) using manufacturer’s protocol. Cells were placed in ice and analyzed using a Beckman Coulter Cytoflex.

### Lentivirus-Mediated shRNAs

All shRNA vectors were obtained from the RNA Interference Screening Facility of Dana-Farber Cancer Institute unless indicated. The target sequences and/or RNAi Consortium clone IDs for each shRNA are as follows: shGFP: GCAAGCTGACCCTGAAGTTCAT (Addgene plasmid #30323); shKras#1: TRCN0000033260 (GAGGGCTTTCTTTGTGTATTT); shKras#2: TRCN0000033262 (gift from Dr. WC Hahn and Dr. AJ Aguirre CCTATGGTCCTAGTAGGAAAT); shmyc#1: TRCN0000039640 (Sigma, CAGTTGAAACACAAACTTGAA); shmyc#2: TRCN0000039639 (sequence: CCCAAGGTAGTTATCCTTAAA); shRPIA#1: TRCN0000049410 (GAATTGGAAGTGGTTCTACAA); shRPIA#2: TRCN0000369927 (GGGAGTTAAATCCAGTCTTAT); shDHODH#1: TRCN0000025839 (Sigma, CGATGGGCTGATTGTTACGAA); shDHODH#2: TRCN0000025868. (Sigma, GTGAGAGTTCTGGGCCATAAA). Lentivirus were produced using 293 T cells as previously described^11^. For rescue studies with mouse RPIA, RPIA cDNA (Vector Builder, VB150325–10041, NM_009075.2) was cloned into the doxycycline-inducible pInducer20 plasmid (Addgene, plasmid #44012) and introduced in MiaPaCa2 cells using the same lentiviral system described. Overexpression of RPIA was induced with 200 nM of doxycycline 48 h before knock-down.

### CRISPR

Gene editing was performed as described previously^46^. Briefly, the sequence targeting exon 4 of human RPIA (GGTGTCGATCCAGATCACTGAGG) was introduced into sgRNA oligos. The sgRNA oligos were cloned into the Cas9 vector (Addgene, plasmid #51760) and transformed into Stbl3 bacteria (Invitrogen, C7373-03). Colonies were isolated and analyzed by PCR to confirm correct insertion of sgRNA. After sequence validation, viruses were produced using 293 T cells. After selection, pools of cells were used to assess growth and clonogenic ability.

### Quantitative PCR

mRNA was isolated using TRIzol (Invitrogen) per protocol and DNAse treated. Reverse transcription was performed with 2 µg of RNA using oligo-dT and MMLB-HP reverse transcriptase (Epicentre). Quantitative RT-PCR was performed using SYBR Green in a Mx3000PTM machine (Stratagene). All reactions were performed in triplicate and actin was used as internal control. Sequences for primers are as follows: Myc_Fw: GGCTCCTGGCAAAAGGTCA, Myc_Rv: AGTTGTGCTGATGTGTGGAGA, RPIA_Fw: TCTGAACCTCGTCTGTATTCCC, RPIA_ Rv: ACTGAGGGTCAAGCCATACTG, RPE_Fw: AACCAGGAACCTCAGTTGAGT, RPE_Rv: CACTGTCATAACCAAGGCCAT, HK1_Fw: CCAACATTCGTAAGGTCCATTCC, HK1_Rv: CCTCGGACTCCATGTGAACATT, HK2_Fw: GAGCCACCACTCACCCTACT, HK2_Rv: CCAGGCATTCGGCAATGTG, LDHA_Fw: TTGACCTACGTGGCTTGGAAG, LDHA_Rv: GGTAACGGAATCGGGCTGAAT, G6PD_Fw: TTCCATCAGTCGGATACACACA, G6PD_Rv: ATGAAGGTGTTTTCGGGCAGA, PGLS_Fw: CATCCCGGTTTTCGACCTG, PGLS_Rv: TCGGGGAGTCACTGATGGG, ACTIN_Fw: CATGTACGTTGCTATCCAGGC, ACTIN_Rv: CTCCTTAATGTCACGCACGAT.

### Western blot analysis

Cells were scraped on ice in RIPA buffer containing protease and phosphatase inhibitors. Proteins were separated in 4–20% SDS-PAGE gels (Biorad, 4561095) and transferred to polyvinylidene difluoride (PVDF) membranes (Bio-Rad). Membranes were blocked in 5% dry milk in Tris Buffered Saline with Tween 20 (TBST 1×). Incubation with primary antibodies was performed at 4 °C overnight or for 1.5 h at room temperature. Membranes were washed with TBST 1×, incubated with peroxidase-conjugated secondary antibody for 1 h and developed using the Enhanced Chemiluminescence (ECL) Detection System (Thermo Scientific). Antibodies are as follows: pAkt (Ser473, Cell Signaling #4060 S, 1:1000), Akt (Cell Signaling #9272, 1:1000), pErk1/2 (Thr202/Tyr204, Cell Signaling #4376, 1:1000), Erk1/2 (Cell Signaling #9102, 1:1000), c-Myc (D84C12, Cell Signaling #5605 S, 1:1000), DHODH (Protein Tech 14877-1-AP, 1:1000), Kras (Santa Cruz F234 sc-30, 1:100), RPIA (Abcam Ab86123, 1:1000), β-Actin (Sigma A5441, 1:3000), Anti- rabbit IgG (H1L) HRP conjugate (Thermo 31460, 1:3000); anti-mouse IgG(H1L)HRPconjugate (Promega W4021, 1:7000). Uncropped blots are shown in Supplementary Figures 8–9.

### Metabolism studies

OCR and ECAR measurements were performed using an XF-96 Instrument (Seahorse Biosciences). For shKras cells, 20,000 cells were plated in quadruplicate the day before and measures were performed 24 h after plating. For AZD8330 treatment, 7000 cells were plated in 100 µl of media. 6 h after plating, AZD8330 was added to a final concentration of 50 nM and left for 72 h. For 24 h treatment, drug was added the day before measuring. In all cases, the day of measure, media was replaced with reconstituted DMEM (25 mM glucose, 4 mM glutamine, no sodium bicarbonate and pH adjusted to 7.4) and cells were incubated at 37 C for 30 min in a CO2-free incubator. Mitochondrial stress test was performed as described elsewhere^9^. OCR and ECAR were normalized to cell number as determined by crystal violet at the end of the experiment. For glucose uptake and lactate production measurement, cells were seeded in 12-well plates in triplicate for 48 h. Glucose and lactate concentrations were measured in fresh and spent medium using a Yellow Springs Instruments (YSI) 2900. Glucose data is presented as net decrease in concentration, and lactate as net increase in concentration after normalization to cell number. Relative PPP arm activity was performed as described elsewhere^19^.

### Metabolomics

Steady state metabolomics experiments were performed as previously described^9,10^. Briefly, shKras cells were plated in 6 cm plates in biological triplicate (DMEM, 25 mM glucose, 4 mM glutamine, 10% FBS). Media was changed two hours before metabolite collection. For AZD8330 treatment, cells were plated and media was refreshed the next day with drug/DMSO. Metabolite collection was performed 24 or 72 h after drug addition. Experiments were performed in 25 mM glucose/4 mM glutamine and 10 mM glucose/2 mM glutamine. For N15-Glutamine tracing tracing experiments, cells were plated as described and pre-treated for 4 h with AZD8330 (50 nM) in 10 mM glc/2 mM glutamine DMEM. After that, media was refreshed to stable isotope-labeled glutamine (Amide-^15^N) DMEM containing 10% dialysed FBS and 10 mM glucose for 20 h. Media was refreshed 2 h before metabolite collection with media containing the labelled metabolite and drug, as described. Metabolite fractions were normalized to cell number obtained in a parallel 6 cm plate. 13C-isotope-labeled glucose (13 C) tracing experiments were performed as described elsewhere^19^. For tissue metabolomics, metabolite fractions were normalized to tissue weight.

### Xenografts

Xenograft studies were performed as described previously^9,10^. Briefly, 2 × 10^6^ Panc-1 cells were injected in the flanks of nude female mice (Taconic ncrnu-f, 6 weeks, 10 mice per group) under Dana-Farber Cancer Institute IACUC protocol 10–055. Tumor volume was measured twice a week based on caliper measurements of tumor length and height as volume = (length × width^2^)/2. A second investigator performed the tumor measurements blinded to treatment groups. Mice were separated into two groups matched for tumor volume. Treatment was initiated when the total volume per group was 200 mm^3^. The group to receive the treatment vs. control was randomly determined. Brequinar was dissolved in 70% PBS 1 × /30% PEG-400 and pH was adjusted to 7.00 with NaOH. Mice were injected once every other day for 4 weeks at 50 mg/kg with BQ or vehicle. For toxicity experiments, mice were injected for two weeks and at end-point, blood was analyzed using an Advia 2120i.

### Histology

Tumors were processed as previously described^12^. Antibodies used for immunostaining are as follows: Cleaved caspase-3 (D175) (Cell Signaling Technology 9661; 1:500), Ki67 (Abcam Ab15580; 1:400), α-SMA (Dako, M0851, 1:500). Sections from 9 (vehicle) and 8 tumors (treatment group) were examined microscopically, and five or more representative fields from each slide were quantified. Proliferation index was calculated as number of positive cells vs. total.

### Chemicals

^15^N-labelled glutamine (Cambridge Isotope Laboratories NLM-557-0.5), D-glucose (Sigma G7528), L-glutamine (Sigma G3126), AZD8330 (Selleckchem, S2134), trametinib (Selleckchem, S2673), GDC0941 (Selleckchem, S1065), leflunomide (Sigma L-5025), brequinar sodium salt (Sigma, SML0113), uridine (Sigma, U3003), inosine (Sigma, I4125), mitochondrial stress kit (Seahorse 101706-100), VECTASTAIN Elite ABC Kit (pk-6100; Vector Labs), DAB (sk-4100; Vector labs).

### Statistical analysis

Statistical analysis was done using GraphPad PRISM software. No statistical methods were used to predetermine sample size. For comparisons between two groups, Student’s *t*-test (unpaired, 2-tailed) was performed. Groups were considered different when *p* < 0.05.

## Electronic supplementary material

Supplemental Information

## Data Availability

The authors declare that all the data supporting the findings of this study are available within the article and its Supplementary Information files and from the corresponding author upon reasonable request.
