# Supplementary material for: Oncogenic KRAS supports pancreatic cancer through regulation of nucleotide synthesis
Source: Nat Commun. 2018 Nov 23;9:4945. doi: 10.1038/s41467-018-07472-8 (PMC6251888; doi:10.1038/s41467-018-07472-8)
Supplement: Supplementary file 1 — Supplemental Information [file 41467_2018_7472_MOESM1_ESM.pdf]

Oncogenic KRAS supports pancreatic cancer through regulation of nucleotide synthesis

Santana-Codina et al.

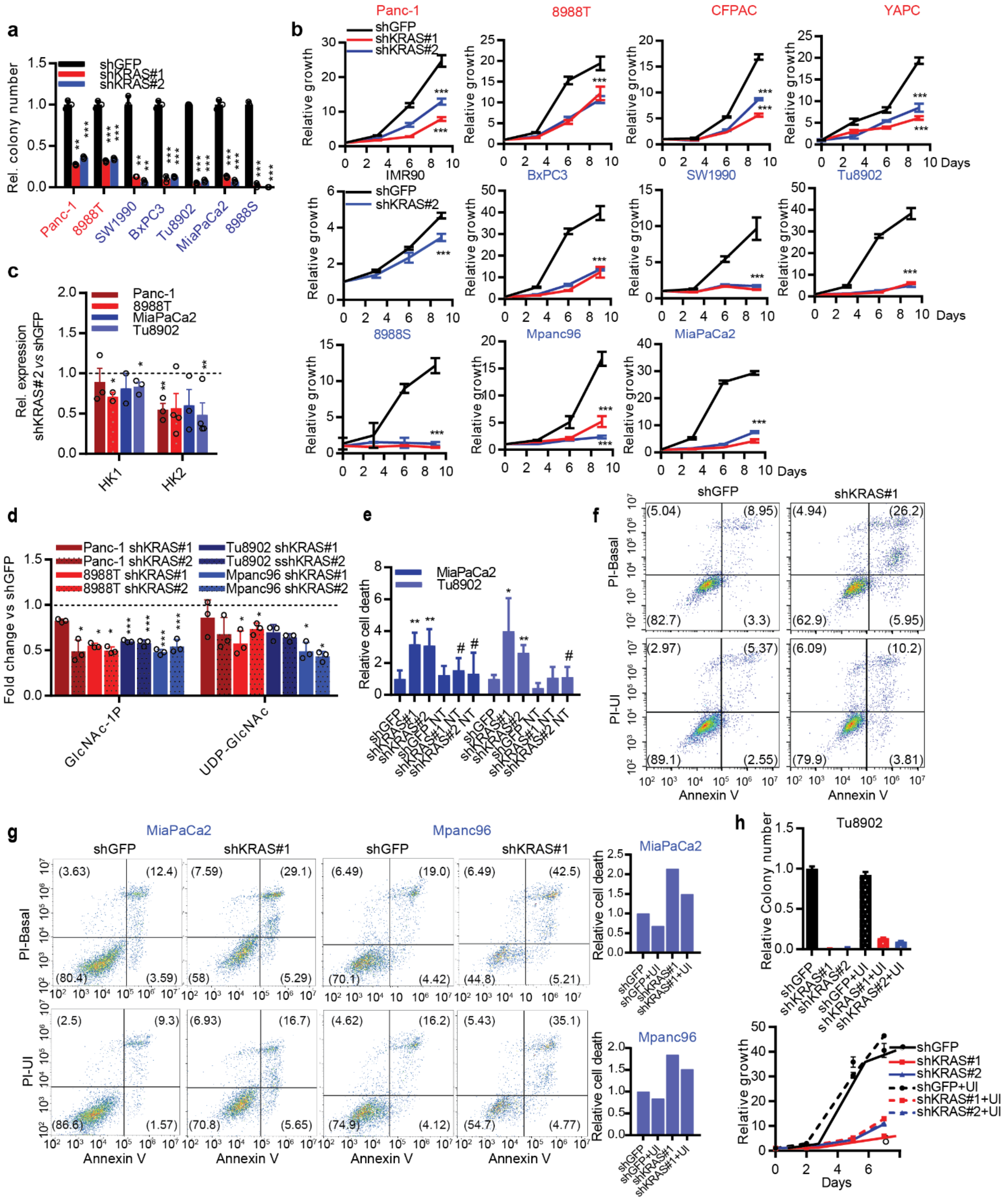

Supplementary Figure 1. **KRAS effects on metabolism, growth and survival of PDAC cells: a role for nucleotides. a,** Relative clonogenic growth in a panel of PDAC cells expressing a control shRNA (shGFP) or two independent shRNAs targeting KRAS. Error bars represent s.d. of triplicate wells from a representative experiment (8988T, MiaPaCa2 n=5; Panc-1, Tu8902, 8988S n=3; SW1990, BxPC3 n=2) experiments. **b,** Relative growth of PDAC cells depleted of KRAS expression using two different lentiviral shRNAs. Values normalized to Day 0. Error bars represent s.d. of triplicate wells of a representative experiment (8988T, MiaPaCa2, Panc-1, Tu8902, Mpanc96, CFAPC, YAPC n=3; BxPC3, SW1990, 8988S, IMR90 n=2). **c,** Relative mRNA levels of glycolytic genes after KRAS depletion (shKRAS#2 vs. shGFP), normalized to β-actin. Error bars represent s.e.m of independent experiments. **d,** Fold change of HBP intermediates after KRAS inhibition, each shRNA relative to its corresponding shGFP. Error bars represent s.d. of n=3 technical replicates from independently prepared samples from individual wells. Significance determined for each shRNA relative to its corresponding shGFP. n=3. GlcNAc-1P, N-Acetyl-glucosamine-1-phosphate; UDP-GlcNAc,

UDP-N-Acetyl-glucosamine. **e,** Nucleotide supplementation rescues KRAS inhibition-induced cell death. Viability was measured by trypan-blue exclusion assay after 48h in MEM or MEM with nucleosides. Error bars represent s.e.m of 3 averaged experiments (basal data represented in Figure 1a). **f,** Flow cytometry analysis of cell death in Tu8902 after KRAS depletion and supplementation with nucleotides: Uridine (U) and Inosine (I) for 48h at 1 mM respectively (one representative of 5 experiments, shown as a bar graph in Figure 1f). **g,** Cell death analyzed by flow cytometry after KRAS depletion in MiaPaCa2 and Mpanc96 cells in media supplemented with U (uridine) and I (inosine) for 48h at 1 mM, respectively (one representative of 4 (MiaPaCa2) or 3 (mPanc96) experiments. **h,** Clonogenic growth (left) and relative proliferation (right) of Tu8902-shKras after addition of Uridine (U) and Inosine (I), 1 mM respectively. For all panels, significance determined with *t-test*. *p<0.05, **p<0.01, ***p<0.001.

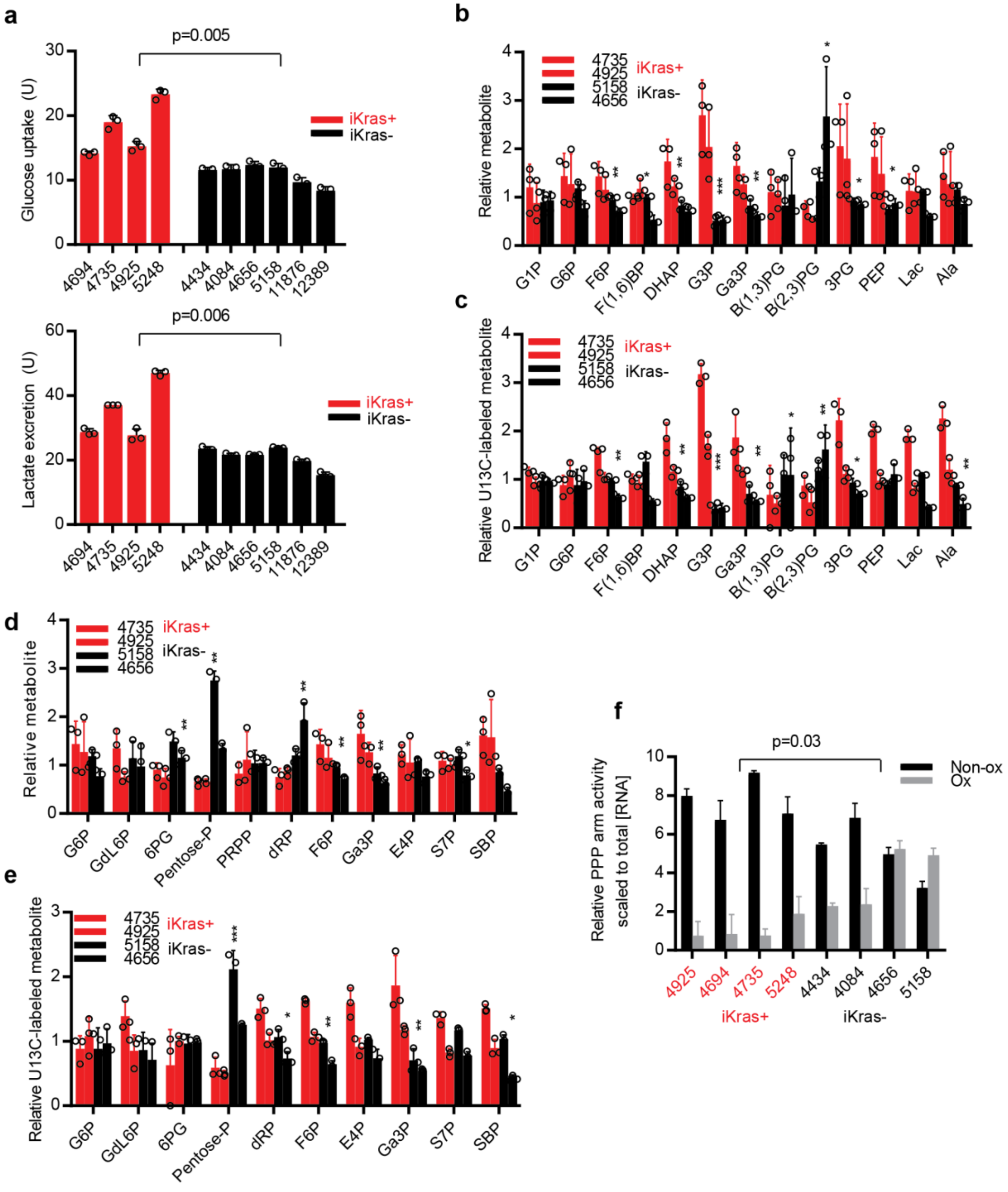

Supplementary Figure 2. Nucleotides mediate resistance in an iKras escaper murine cell model. a, Relative changes of glucose (top) or lactate (bottom) levels in the culture medium from iKras+ or iKras- cells were measured and normalized to cell number. Error bars represent s.d. of n=3 technical replicates from a representative experiment (n=3). **b,** Fold change of glycolytic intermediates in iKRas+ (4735, 4925) vs. iKras- (5158, 4656) murine cells. Error bars represent s.d. of the mean. n=3. **c,** Tracing experiments in iKRas+ (4735, 4925) and iKras- (5158, 4656) cells in medium containing uniformly 13C-isotope-labeled glucose (13C) for 16h to label incorporation into glycolysis. **d,** Fold change of PPP intermediates in iKRas+ (4735, 4925) vs. iKras- (5158, 4656) murine cells. Error bars represent s.d. of the mean. n=3. **e,** Tracing experiments in iKRas+ (4735, 4925) and iKras- (5158, 4656) cells in medium containing stable isotope-labeled glucose (13C) for 16h to label incorporation into glycolysis and PPP intermediates. **f,** Flux through the PPP as determined by 14C-glucose–derived carbon into RNA. Cells were treated with 1-14C- or 6-14C-glucose for 16h. Scintillation counts were performed on purified RNA. Data are presented as relative flux through the non-oxidative (1-14C) and oxidative arm (6-14C – 1-14C). For all panels, significance determined with *t-test*. *$p<0.05$, **$p<0.01$, ***$p<0.001$.

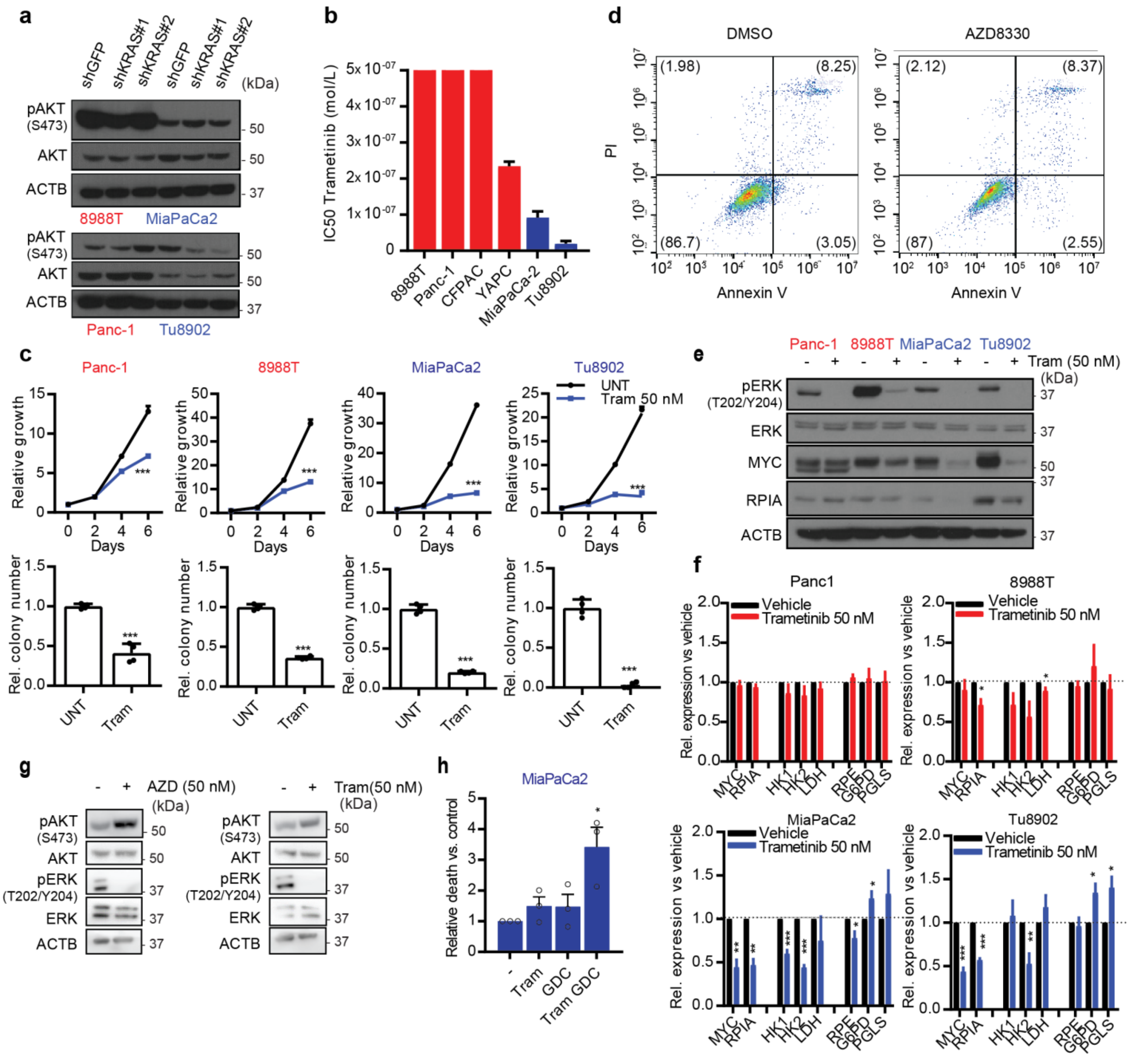

Supplementary Figure 3. Mechanisms of resistance to MEK inhibitors: validation with trametinib. a, pAKT and AKT expression after KRAS depletion in the panel of PDAC cells depicted in Figure 2A. **b,** IC_{50} measurements (y-axis, IC_{50} mol/L) of PDAC cells (x-axis) treated with the MEK inhibitor Trametinib. s.e.m. (8988T, Panc-1 n=5; CFPAC, MiaPaCa2 n=3; YAPC, Tu8902 n=2). **c,** Relative growth (top) and colony formation (bottom) of PDAC cells treated with Trametinib (50 nM). Colony number normalized to untreated cells (error bars indicate s.d. of 2 technical replicates in 2 independent experiments). Growth values normalized to Day 0 (error bars represent s.d. of quadruplicate wells of a representative experiment). **d,** Flow cytometry analysis of cell death in Tu8902 cells treated with DMSO or AZD8330 (50 nM) for 72h. Annexin V-FITC and propidium iodide were used to assess the percentage of necrotic and apoptotic cells (one representative of 3 experiments). **e,** Relative expression of proteins was assessed by western blot in human PDAC cells treated with Trametinib (50 nM) for 16h. **f,** Relative mRNA levels of glycolytic and PPP genes in Panc-1, 8988T, MiaPaCa and Tu8902 cells after trametinib treatment (50 nM) for 16h. All values normalized to β-actin and relative to DMSO treated cells. Error bars represent s.e.m of independent experiments (Panc-1, Tu8902 n=4; MiaPaCa2, 8988T n=3). **g,** Relative expression of proteins was assessed by western blot in MiaPaCa2 cells treated with AZD8330 (50 nM) and Trametinib (50 nM) for 16h. **h,** Cell death analyzed by flow cytometry in MiaPaCa2 cells treated with trametinib (50 nM), GDC0941 (2 µM) or their combination for 72h. Bars represent relative fold change in cell death *vs.* shGFP (error bars show s.e.m of 3 independent experiments). For all panels, significance determined with *t-test*. *p<0.05, **p<0.01, ***p<0.001.

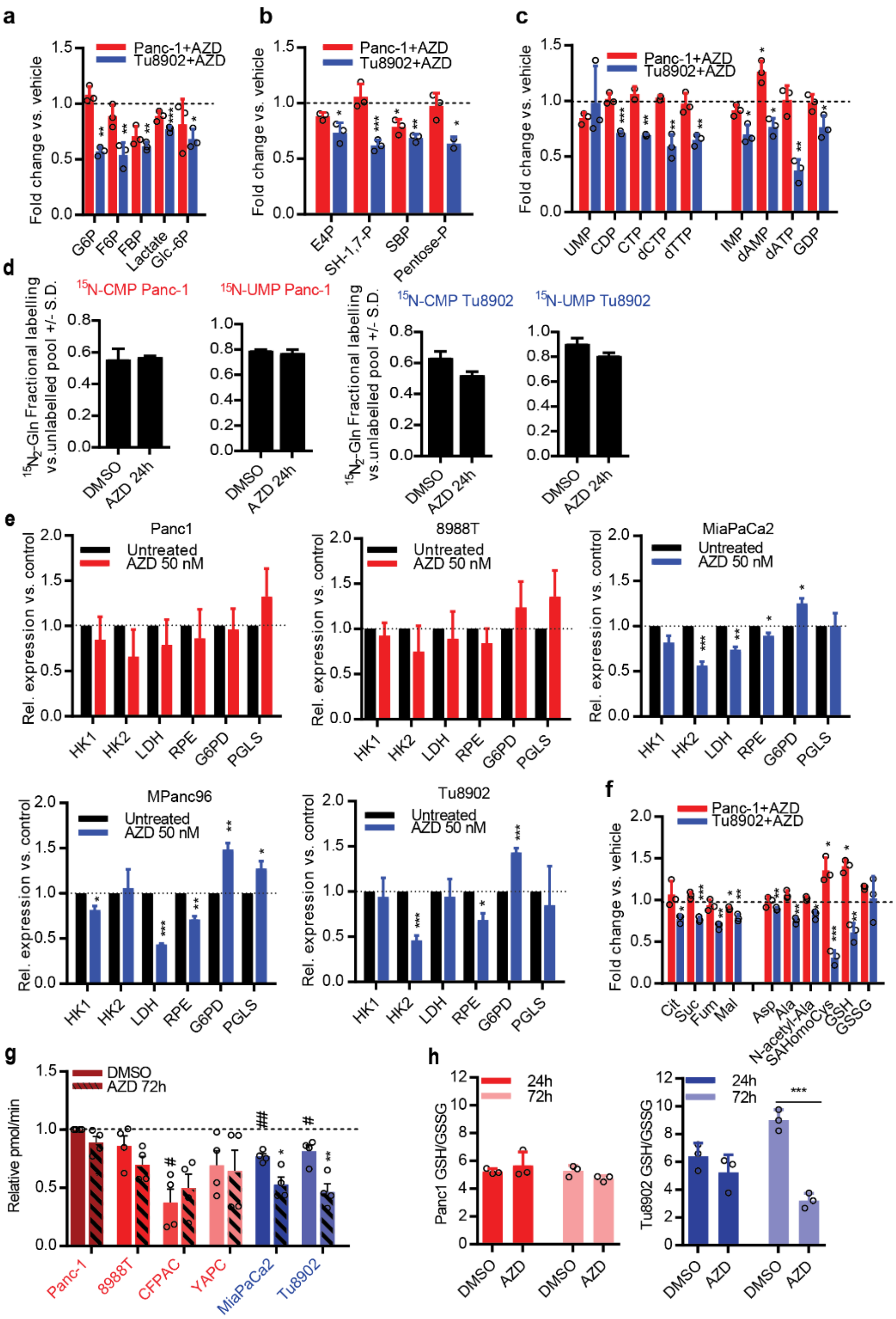

Supplementary Figure 4. Metabolic consequences of MEK inhibition. - For all metabolomics experiments in this figure, cells were treated with AZD8330 (50 nM) for 24h in media containing glucose 10 mM and glutamine 2 mM. Values are represented as fold change *vs*. DMSO treated cells. Error bars represent s.d. of n=3 technical replicates from independently prepared samples from individual wells. Fold change of metabolites in the glycolytic and HBP pathway **(a)**, PPP **(b),** pyrimidines/purines **(c). d,** Tracing experiments in Panc-1 and Tu8902 cells treated with AZD8330 (50 nM) in medium containing stable isotope-labeled glutamine (Amide-15N) for 24h to label incorporation into the pyrimidine ring. **e**, Relative mRNA levels of glycolytic/PPP genes after AZD8330 treatment (50 nM, 16 h). All values normalized to β-actin and relative to DMSO treated cells. Error bars represent s.e.m of pooled independent experiments (8988T n=4; Panc-1, MiaPaCa2, MPanc96, Tu8902 n=3). **f**, Fold change of metabolites in the TCA/transaminase/redox pathways. **g,** Analysis of oxygen consumption rate (OCR) normalized to cell number (crystal violet). All values normalized to Panc-1 control (DMSO), error bars ± s.e.m of 4 independent experiments (* shows comparison to each cell line's control (DMSO), # shows comparison to Panc1-DMSO). **h,** GSH/GSSG ratio after AZD8330 treatment for 24 and 72h in DMEM (glucose 25 mM, glutamine 4 mM) in Panc-1 (left) and Tu8902 (right) cells, Error bars represent s.d. of n=3 technical replicates from a representative experiment. For all panels, significance determined with *t-test*. *p<0.05, **p<0.01, ***p<0.001.

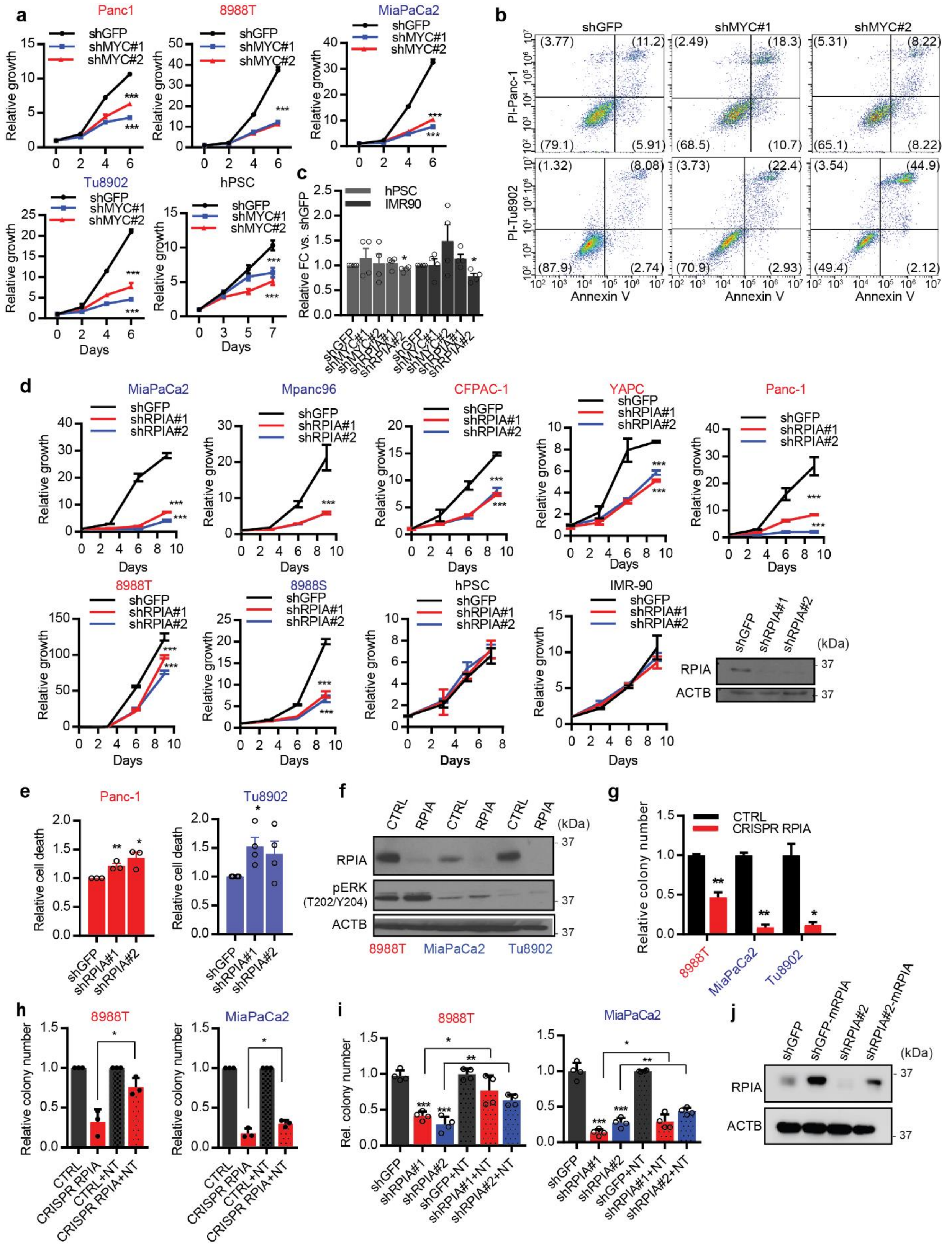

Supplementary Figure 5. MYC and RPIA inhibition decrease proliferation and clonogenic growth in PDAC cells. a, Relative growth after MYC depletion with two independent shRNAs. Values normalized to day 0. Error bars represent s.d. of triplicate wells of a representative of 4 (hPSC), 3 (8988T, 8902) or 2 (Panc-1, MiaPaCa2) experiments. **b,** Flow cytometry analysis of cell death in Panc-1 and Tu8902 after MYC depletion (48h). One representative experiment of bar graph shown in Figure 4b. **c,** Cell death analyzed by flow cytometry after MYC/RPIA depletion in hPSC and IMR90 cells. Bars represent relative fold change in cell death *vs.* shGFP (error bars show s.e.m of 4 independent experiments, shGFP data represented also in Supplementary Figure 6c). **d,** Relative growth of PDAC cells expressing a control shRNA (shGFP) or two independent shRNAs targeting RPIA. Error bars

represent s.d. of triplicate wells from a representative of 4 (hPSC), 3 or 2 (8988S, IMR90) experiments. (Right) Representative immunoblot of RPIA knock-down in IMR-90 cells. **e,** Cell death analyzed by flow cytometry after RPIA depletion in Panc-1 and Tu8902 cells. Bars represent relative fold change in cell death *vs.* shGFP (error bars show s.e.m of three (Panc-1) or four (Tu8902) experiments). **f,** Immunoblotting of human PDAC cell lines were RPIA was depleted using an sgRNA sequence. **g,** RPIA extinction by CRISPR targeting reduced clonogenic growth of PDAC cells. Relative colony number normalized to control (CTRL) sequence. Depicted is one representative experiment of 3 (or 2 for Tu8902), average ± s.d. This data is included in the bar graph shown in Figure S5h. **h,** RPIA was targeted using a CRISPR sequence (sgRNA) and clonogenic growth was compared to a control sequence (CTRL). Colony number for CRISPR RPIA was normalized to the corresponding control in each condition (MEM or MEM with nucleosides). Error bars represent s.e.m of n=3 independent experiments. *p<0.05, *t*-test. **i,** Relative clonogenicity of shGFP or shRPIA cells (8988T,left, MiaPaCa2, right) cultured in MEM or MEM containing nucleosides (0.04 mM). Each shRNA is normalized to its corresponding control in each condition (with or without nucleosides). Error bars indicate s.d. of 2 technical replicates in 2 independent experiments. **j,** Immunoblotting of MiaPaCa2-mRPIA cells after RPIA depletion and induction of the RPIA construct with doxycycline (200 ng/ml, shRPIA#2-mRPIA). For all panels, significance determined with *t-test*. *p<0.05, **p<0.01, ***p<0.001.

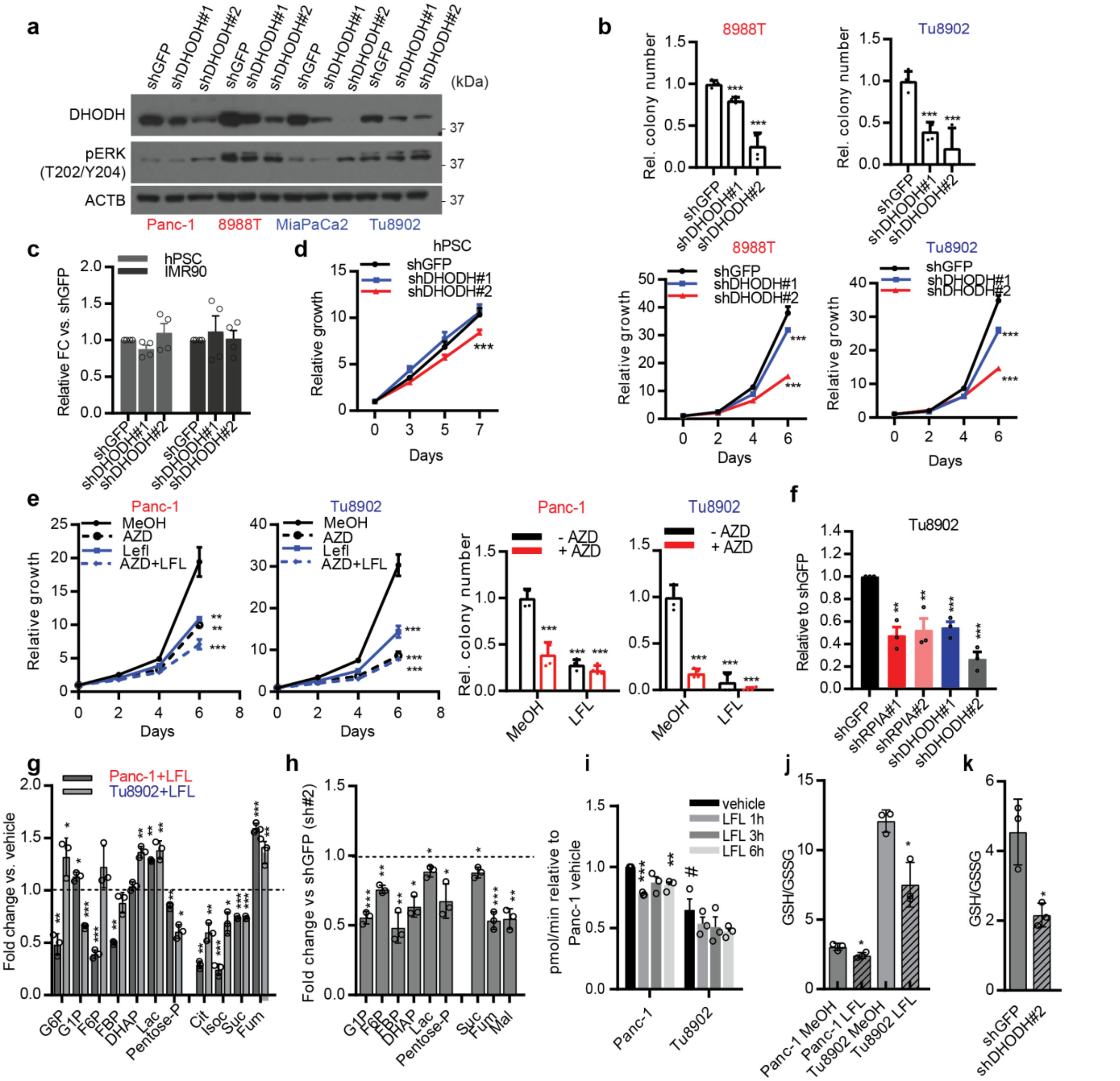

Supplementary Figure 6. Genetic and pharmacological DHODH inhibition impairs proliferation of PDAC cells. **a,** Immunoblotting of PDAC cells after DHODH depletion with two different lentiviral shRNAs and a control sequence (shGFP). **b,** Clonogenic growth (top) and relative proliferation (bottom) of 8988T and Tu8902 cells was assessed after DHODH depletion with two different shRNAs. Relative colony number is normalized to shGFP control (error bars indicate s.d. of 2 technical replicates in 2 independent experiments). Growth values normalized to Day 0 (error bars represent s.d. of quadruplicate wells of a representative experiment). **c,** Cell death analyzed by flow cytometry after DHODH depletion in hPSC and IMR90 cells. Bars represent relative fold change in cell death *vs.* shGFP (error bars show s.e.m of 4 independent experiments, shGFP data represented also in Supplementary Figure 5c). **d**, Relative proliferation of hPSC cells treated after DHODH depletion (error bars indicate s.d. of 3 technical replicates in 4 independent experiments). **e,** Relative proliferation (left) and clonogenic growth (right) of Panc-1 and Tu8902 cells treated with leflunomide (50 μM). Relative colony number is normalized to shGFP control (error bars indicate s.d. of 2 technical replicates in 2 independent experiments). Growth values normalized to Day 0 (error bars represent s.d. of quadruplicate wells of a representative experiment). **f,** Cells depleted of RPIA and DHODH were seeded in low-adherent plates and their capacity of growing as 3D spheroids in suspension was assessed (error bars show s.e.m of 3 independent experiments). **g and h,** LC-MS/MS metabolomic analysis of glycolytic and TCA cycle intermediates in Panc-1 and Tu8902 cells treated with leflunomide (100 μM, 16h) **(g)** or Panc-1 shDHODH#2 cells **(h).** Fold change relative to vehicle (methanol in **g**) or shGFP **(h)**. Error bars represent s.d. of n=3 technical replicates from independently prepared samples from individual wells. Cit, citrate; Isoc, isocitrate; α-keto, α-ketoglutarate; Suc, succinate; Fum, fumarate. **i,** Leflunomide (50 μM) short-term treatment effects on OCR in Panc-1 and Tu8902 cells, LFL, leflunomide.

Error bars show s.e.m of 3 independent experiments (* shows comparison to each cell line's control (DMSO), # shows comparison to Panc1-DMSO). **j,** Leflunomide (100 μM, 16h) decreases the ratio of reduced-to-oxidized glutathione (GSH/GSSG) in Panc-1 and Tu8902 cells. **k,** DHODH ablation decreases the ratio of reduced-to-oxidized glutathione (GSH/GSSG) in Tu8902 cells. For all panels, significance determined with *t-test*. *p<0.05, **p<0.01, ***p<0.001.

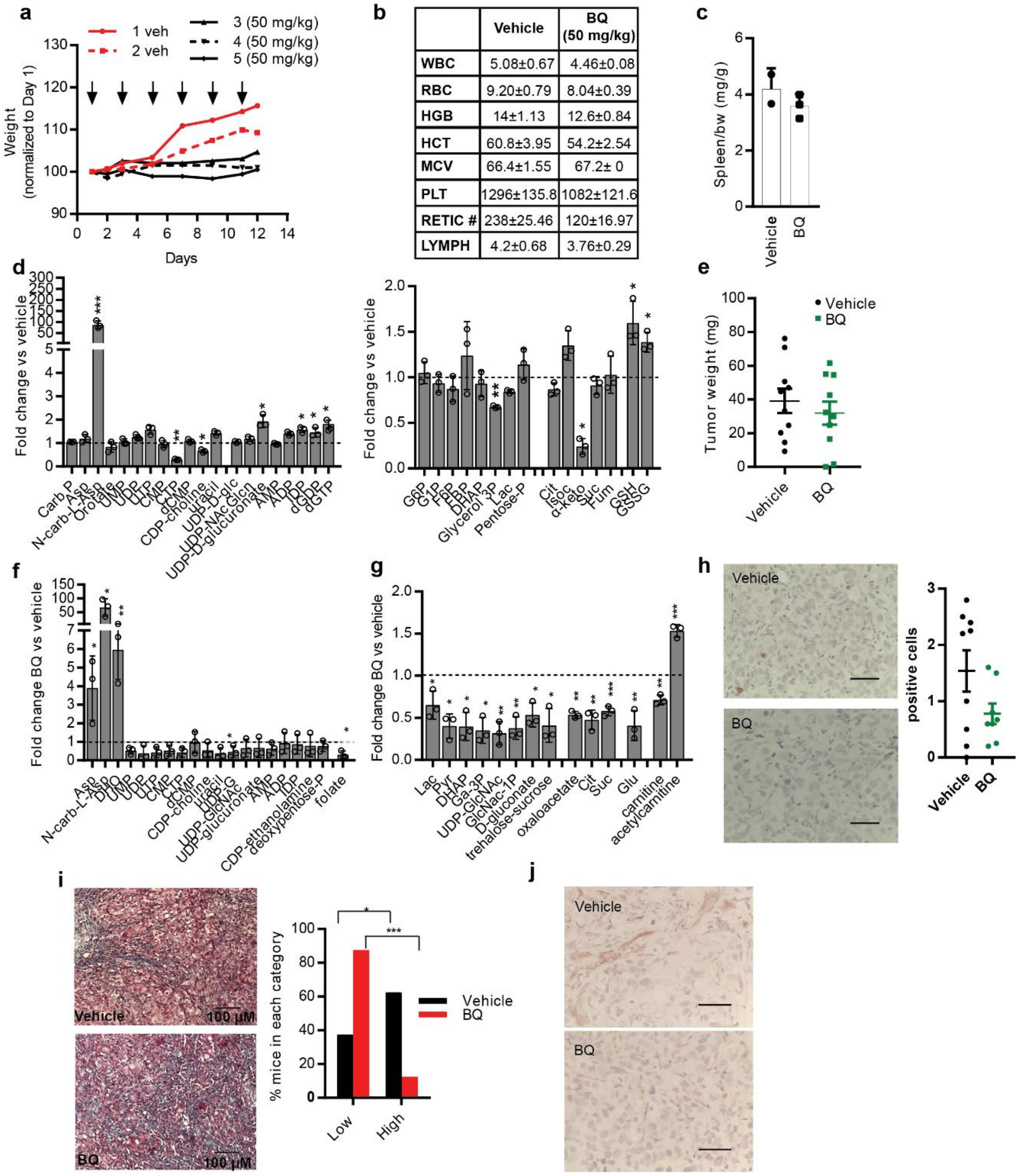

Supplementary Figure 7. Brequinar inhibits DHODH with minimal toxicity *in vivo*. a, Pilot experiment with a 50 mg/kg dose every other day. BQ-treated mice maintain body weight. **b,** Blood from 2 (vehicle) and 3 (BQ)-treated mice for 2 weeks was withdrawn by retro-orbital bleed and analyzed in an Advia machine (1 to 4 dilution in PBS). WBC (White Blood Cells), RBC (Red Blood Cells), HGB (Hemoglobin), HCT (Hematocrit), MCV (Mean Corpuscular Volume), PLT (platelet), RETIC (reticulocyte), LYMPH (lymphocytes). **c,** Spleen was weighed at the end of the pilot experiment and spleen weight was normalized to total body weight. **d,** LC-MS/MS metabolomic analysis of pancreata extracted 4h after dosing. Error bars represent s.d. of n=3 mice per group. **e,** Tumor weight in the vehicle and BQ-treated group of mice (10 mice per group). Error bars represent s.e.m. **f,** LC-MS/MS metabolomic analysis of tumors from mice dosed with BQ or vehicle twice. Error bars represent s.d. of n=3 tumors per group. **g,** LC-MS/MS metabolomics of tumors from mice dosed with BQ or vehicle 4 weeks. Error bars represent s.d. of n=3 tumors per group. **h,** BQ treatment does not induce cleaved caspase 3 in treated mice vs. vehicle (left: representative field (20X) of 5 quantified fields per animal (8 mice per group), scale bar=50 µm, right: quantification of positive cells per field, error bars represent s.e.m.). **i,** Trichrome staining of vehicle and BQ-treated mice (left: representative field (10X) of 5 quantified fields per animal (8 mice per group); right: represents intensity of staining 1-4 graded by a pathologist. Tumors were classified as low (1+ and 2+) or high (3+ and 4+) in collagen staining and percentage of mice in each category *vs.* total mice was represented). **j,** Representative field (20X) of 5 quantified fields per animal (8 mice per group) for α-SMA staining (scale bar=50 µm). For all panels, significance determined with *t-test*. *p<0.05, **p<0.01, ***p<0.001.

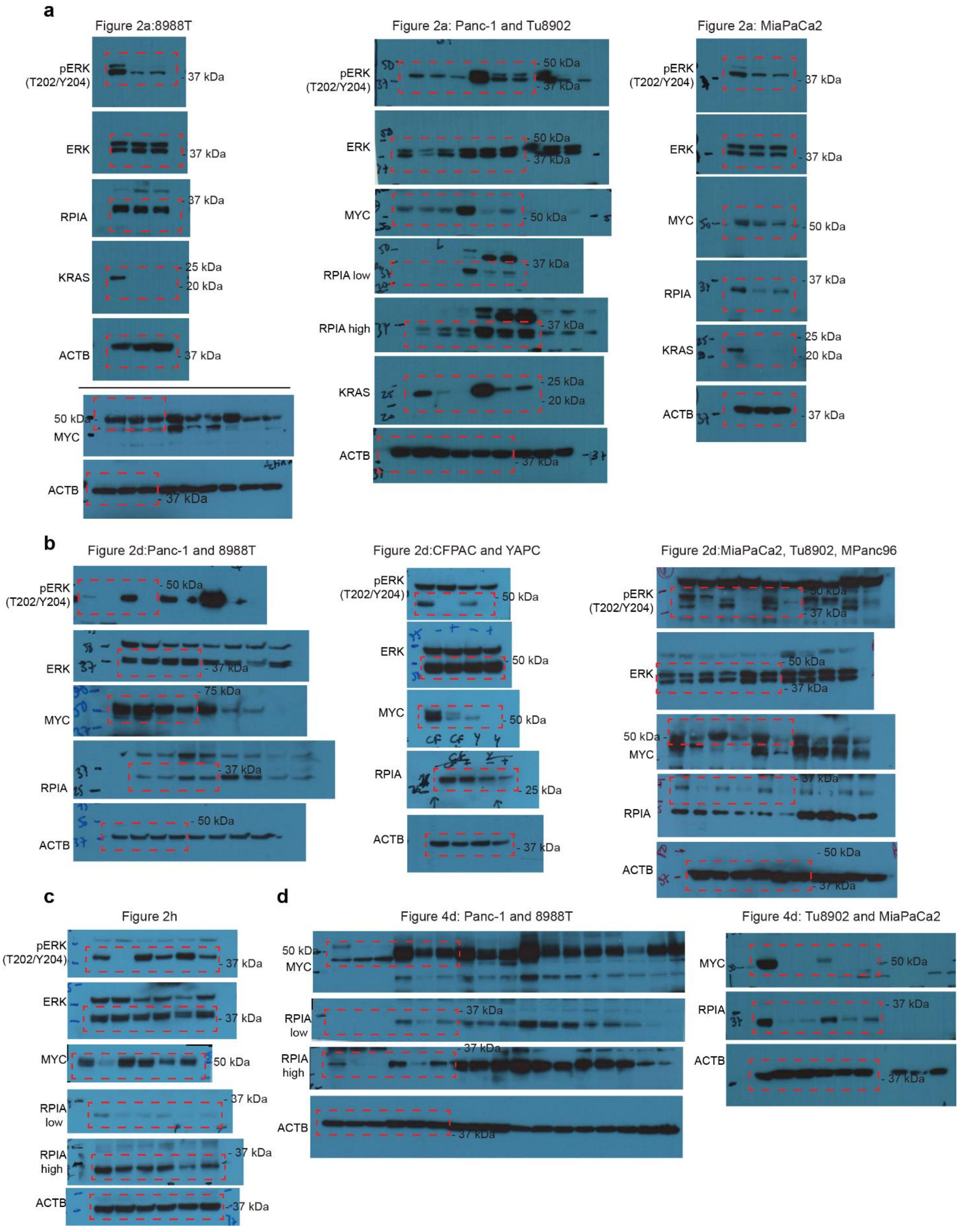

Supplementary Figure 8. Uncropped immunoblots for all data elements shown in main figures. a, Uncropped immunoblots for Fig. 2a: pERK (T202/Y204), ERK, MYC, RPIA, KRAS, ACTB. **b,** Uncropped immunoblots for Fig. 2d: pERK (T202/Y204), ERK, MYC, RPIA, ACTB. **c,** Uncropped immunoblots for Fig. 2h: pERK (T202/Y204), ERK, MYC, RPIA, ACTB. **d,** Uncropped immunoblots for Fig. 4d: MYC, RPIA, ACTB.

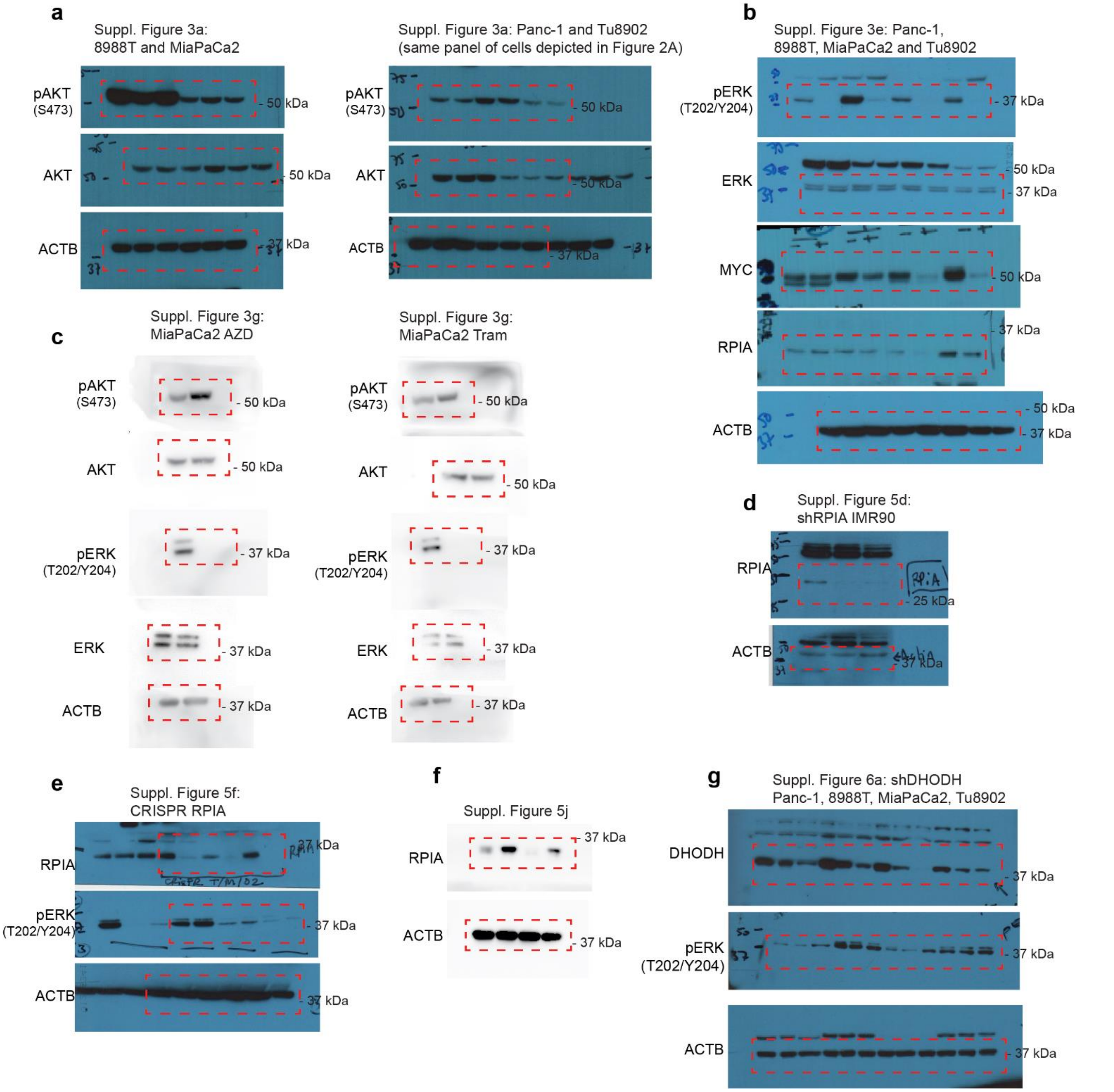

Supplementary Figure 9. Uncropped immunoblots for all data elements shown in Supplementary Figures. a, Uncropped immunoblots for Suppl. Fig. 3a: pAKT (S473), AKT, ACTB. **b,** Uncropped immunoblots for Suppl. Fig. 3e: pERK (T202/Y204), ERK, MYC, RPIA, ACTB. **c,** Uncropped immunoblots for Suppl. Fig. 3g: pAKT (S473), AKT, pERK (T202/Y204), ERK, ACTB. **d,** Uncropped immunoblots for Suppl. Fig. 5d: RPIA, ACTB. **e,** Uncropped immunoblots for Suppl. Fig. 5f: RPIA, pERK (T202/Y204), ACTB. **f,** Uncropped immunoblots for Suppl. Fig. 5j: RPIA, ACTB. **g,** Uncropped immunoblots for Suppl. Fig. 6a: DHODH, pERK (T202/Y204), ACTB.
